# Health-related regional and neighborhood correlates of sexual minority concentration: A systematic review

**DOI:** 10.1371/journal.pone.0198751

**Published:** 2018-06-27

**Authors:** Joseph G. L. Lee, Thomas Wimark, Kasim S. Ortiz, Kerry B. Sewell

**Affiliations:** 1 Department of Health Education and Promotion, College of Health and Human Performance, East Carolina University, Greenville, North Carolina, United States of America; 2 Department of Human Geography, Stockholm University, Stockholm, Sweden; 3 Department of Sociology & Criminology, University of New Mexico, Albuquerque, New Mexico, United States of America; 4 Laupus Health Sciences Library, East Carolina University, Greenville, North Carolina, United States of America; Institute of Tropical Medicine (NEKKEN), Nagasaki University, JAPAN

## Abstract

**Background:**

A growing literature explores spatial patterns of regional and neighborhood correlates of sexual minority (e.g., lesbian, gay, bisexual) concentration. Such patterns have implications for health and wellbeing if there are differences in health-promoting or health-hindering resources in neighborhoods or regions. We conducted a systematic review to assess sexual minority concentration in relation to area unit characteristics.

**Methods:**

We included only records published after 1973 and made no exclusions by geography or language. We searched 11 databases (Academic Search Complete, CINAHL, Embase, GeoBase, GeoRef, LGBT Life, PsycINFO, PubMed/MEDLINE, Scopus, Sociological Abstracts, Web of Science) on November 19–21, 2016. We searched reference lists of included records. We used the following inclusion criteria: (1) Record is a quantitative study (that is, it uses statistics to describe or associate two or more variables); (2) Record is about (a) migration or internal migration of, (b) area unit selection by, or (c) concentration of sexual minority people (defined by identity, behavior, or attraction); (3) Criterion 2 is linked to the characteristics of regions or neighborhoods (at any spatial scale).

**Results:**

Dual independent coding resulted in 51 records meeting inclusion criteria from the original pool of 5,591. From these records, we identified the 647 reported results linking sexual minority concentration with area unit characteristics. Of these, 132 were unadjusted relationships between sexual minority concentration and four theory-informed domains of neighborhood influence on health. We identified greater concentration of sexual minorities in regions with more resources and in more urban regions. A limited but troubling literature at the neighborhood level suggested potentially higher concentrations of sexual minorities in neighborhoods with fewer resources.

**Conclusions:**

There are substantial gaps in the literature. We discuss the implications of our findings and gaps in relation to key theories of sexual minority health.

**Registration:**

The review was not registered with PROSPERO because it was not eligible for registration at the time of the research project’s initiation due to the outcome of interest.

## Introduction

Where a person lives can influence their health in a myriad of ways [1–8]. Where people live is not random. Opportunities and constraints at the macro level as well as resources and restrictions at the micro level have created and sustained segregation by socioeconomic status and racial/ethnic identity. Economic and social forces have driven migration within countries and regions. Similar forces shape neighborhood selection, preference, and access. Thus, when these economic and social forces differ by identity or class, they can shape the population distribution of exposure to health harming or health promoting neighborhoods [3, 9] and policies [10–12]. Clear evidence suggests residential segregation impacts the health of racial/ethnic minorities, for example [9].

A growing literature extends this work to examine the spatial patterning of sexual minority (e.g., lesbian, gay, and bisexual [LGB]) people. While reviews have mapped the relationship between neighborhoods, ethnic minorities, and health [9] and to a lesser extent the relationship between neighborhoods and sexual minority health [4], we know much less about where sexual minority populations live [13]. In fact, a systematic synthesis of this relationship is missing from the extant literature [14].

Spatial patterns of sexual minority populations are important for understanding the substantial health inequities that exist for sexual minority individuals and for developing population health interventions thereon [14, 15]. If, for example, the average sexual minority individual is living in a region or neighborhood with fewer health-promoting resources, this could contribute to worse health for sexual minority populations. Regional migration may change exposure to policies that influence health, including tax regulations (e.g., state cigarette taxes). For example, the demographic distribution of race and ethnicity in the U.S.A. results in unequal coverage of clean indoor air laws and cigarette tax exposure by race/ethnicity [16, 17]. Differing social climates (e.g., protections from discrimination) could exacerbate minority stress and stigma [18]. Neighborhood access or selection within a region may also influence exposure to other health-related resources and problems. For example, some neighborhoods have more tobacco marketing [19]. Census-tracts with high proportions of same-sex partners are more likely than those with low proportions to have greater amounts of hazardous air pollutants [20]. Living in a neighborhood with a larger visible sexual minority population could lead to decreased, as well as potentially increased, homophobic harassment and violence [21–25]. However, it is difficult for researchers to hypothesize these relationships without knowing correlates of where sexual minority people live.

A substantial amount of research has examined the formation and characteristics of gay and lesbian neighborhoods using largely qualitative approaches. These studies have argued or shown that gay men’s communal history is linked to urban spaces [26] and that LGB individuals migrate to larger cities [27–31], to specific tolerant cities [32, 33], and to specific “safe” enclaves in cities [34, 35, 36]. However, a line of research has questioned these conclusions by arguing that the relationship with urban areas is more complex [37, 38–42].

Thus, we sought to systematically identify and assess the existing peer-reviewed quantitative literature on the association between the concentration of sexual minority individuals and regional- and neighborhood-level characteristics. We conducted a systematic review with four aims to identify: (1) spatial scales at which previous research has examined spatiality and migration of sexual minority individuals, (2) correlates of where sexual minority individuals live or migrate to at larger spatial scales (e.g., regions), (3) correlates of where sexual minority individuals live or migrate to at smaller spatial scales (e.g., neighborhoods), and (4) use of longitudinal data and assessment of race/ethnicity.

## Methods

Note that we use the following terminology: A *study* is a research project from which multiple papers (i.e., *records*) can be published and in which individual *results* are reported. We write generally about sexual minority people for readability. When writing about a specific record, we use more precise terminology (e.g., male same-sex couples, lesbian women). We shorthand ways of measuring the density, rate, count, or percentage of sexual minority people as *concentration*; this should not be confused with the formal definition of concentration used in the segregation literature. Also for readability, we write generally about area units in two categories: We shorthand larger spatial scales as regions and smaller spatial scales as neighborhoods. We define regions as larger area units equal to or larger than the equivalent of a U.S. county (e.g., city, census place, municipality, metropolitan statistical area). We define neighborhoods as smaller area units that, while often imperfect, can provide meaningful information about the health of communities [43–45]. These include the census block (U.S.A.), tract (U.S.A.), data zone (U.K.), dissemination area (Canada), output area (U.K.), and postal code.

Our inclusion criteria were: (1) Record is a quantitative study (that is, it uses statistics to describe or associate two or more variables); (2) Record is about (a) migration or internal migration of, (b) area unit selection by, or (c) concentration of sexual minority (defined by identity, behavior, or attraction) people; (3) Criterion 2 is linked to the characteristics of regions or neighborhoods (at any spatial scale).

One author (KBS), an information specialist/librarian, iteratively designed the search strategy using recommended keywords for sexual minority-related searches [46] in PubMed/MEDLINE and translated controlled vocabulary to other databases. The final PubMed/MEDLINE search is available online (Online Data Repository Link, 10.15139/S3/GVAIWD, https://dataverse.unc.edu/dataverse/SGM-geography). We implemented the search strategy November 19–21, 2016, in 11 academic databases (Academic Search Complete, CINAHL, Embase, GeoBase, GeoRef, LGBT Life, PsycINFO, PubMed/MEDLINE, Scopus, Sociological Abstracts, Web of Science). No language, geography, or date limitations were included in the search. One author (KBS) also searched for published books. We excluded all records published prior to 1973 (the year homosexuality was removed from the Diagnostic and Statistical Manual in the United States). Records were de-duplicated with reference management software and manually. We used Covidence cloud-based software (covidence.org) to manage the coding process. The title/abstract of each record was independently reviewed for inclusion or exclusion by two authors (JGLL, KSO, KBS, TW). Conflicts were moved to full-text review or resolved by discussion. The full text of each included record was then independently reviewed for inclusion or exclusion by two authors (JGLL, KSO, TW). Conflicts in coding were resolved by discussion. We did not calculate reliability in inclusion coding as our goal in dual, independent coding was to be as sensitive as possible in the inclusion process. References in each included record were examined for additional eligible records. A systematic review protocol is available online (Online Data Repository Link, doi:10.15139/S3/GVAIWD).

One author (JGLL or TW) abstracted study characteristics (e.g., setting area unit[s], time, analysis strategy, and results of interest to this review), and a second author (JGLL or TW) then verified and cleaned the evidence table. Each result was classified by its statistical significance at the p>0.05 threshold. Records not in English were only reviewed by one person, and these records were included and abstracted if they met inclusion criteria. One author (JGLL) reviewed included records for risk of bias. To do this, we utilized a four-item index based on the Downs and Black checklist [47]. We could not identify a risk of bias tool that worked for the etiological, descriptive studies included in this review, as most such tools are designed for assessing intervention studies. We selected one item to reflect external validity (“Were the participants asked to participate in the study representative of the entire population from which they were recruited?”) and three items to reflect internal validity (“Were the LGB participants recruited from the same population as the comparison group?”, “Were the LGB participants recruited during the same time period as the comparison group?”, and “Were the statistical tests used to assess the main outcomes appropriate?”). We coded these so a study with a score of zero was highly biased and a study a score of four was minimally biased.

We present all results in an evidence table (Supplemental File 1). We conduct a narrative review of the results and graphically display results using a modified harvest plot [48]. For the harvest plot, we identified results that provided (1) a statistical test with significance and direction, (2) that were not repeated comparisons against a reference category, (3) that were not adjusted for other covariates, (4) that scored a three or four in our risk of bias index, and, (5) that had a clear relevance for health and resources. Thus, we excluded measures such as the age of the housing stock in a neighborhood as it was not clear to us if this was an indicator of valuable historic properties or an indicator of older, distressed housing. We also excluded measures of segregation that simply described the area unit’s segregation as these did not meet our study aims.

To create the harvest plot [48], we utilized SPSS v. 24 (IBM, Chicago, Illinois) to plot the significance and direction of each included result stratified by area unit size, gender, and domains of neighborhood characteristics. That is, the harvest plot presents a count of results by positive, negative, or non-significant effect. We reverse the signs of reported results as necessary to match the harvest plot’s theoretical framework, which is described next.

The categorization of the results for the harvest plots builds on theoretical ideas on how neighborhood characteristics affect individuals’ health and socioeconomic trajectories [1, 3]. In line with Galster’s identification of mechanisms for how neighborhood characteristics matter to human development [1], we use his four top-level categories: Social-interactive, Environment, Geographical, and Institutional, as shown in Table 1. Following Bernard et al. [3], we refer to them as domains instead of mechanisms as most of the variables in the articles are not explicitly conceptualized as mechanisms. Drawing on both Galster and Bernard, we categorize results within the categories as follows: Social-interactive domain refers to social processes in neighborhoods [1]. Under the Social-interactive domain, we locate deprivation (e.g., housing values, poverty and income), social cohesion (e.g., vacant housing, rental housing), and diversity (e.g., race, ethnicity, foreign-born). The Environment domain signifies the human and physical aspects of the neighborhood [1]. In this category, we find variables related to exposure to violence (e.g., hate crimes, crime), toxic exposure (e.g., air pollution), and minority stress (e.g., % voting conservative, lack of other sexual minority populations). The Geographical domain includes factors that can be located at a larger scale than the immediate neighborhood [1]. In this category, we locate the two subcategories; public services (e.g., antidiscrimination legislation/policies) and rurality. The Institutional domain contains decisions and actions by individuals and institutions outside of the neighborhood [1]. In this category, we locate local institutional resources (e.g., schools, parks, theatres, health clinics) and local market actors (e.g., tobacco retailers, health-harming marketing).

**Table 1:** Categorization and example indicators of neighborhood domains of influence on health.

Galster-Bernard Domain	Indicator	Example Measures
Social-interactive	Deprivation	Housing values, creative class, poverty, income
	Lack of social cohesion	Vacant houses, rental housing
	Lack of diversity	Race/ethnicity, foreign-born, language
Environmental	Poor physical environment	-
	Toxic exposure	Air pollution
	Violence/crime	Hate crimes
	Social sources of minority stress	Conservative votes, concentration of other sexual minorities
Geographical	Rurality	Population density, RUCA, RUCC, city size
	Limited public services/protections	Hate crime legislation, laws
Institutional	Limited local institutional resources	Schools, parks, cultural organizations, health clinics, transportation stations
	Harmful local market actors	Tobacco retailers, marketing of health-harming products

The harvest plot’s pattern of results did not differ in a substantive way when stratified by same-sex couples versus sexual minority individuals. We thus report results of aggregated genders, female, and male. Due to the heterogeneity of study designs, time periods, and measures in the literature, we did not conduct a meta-analysis. We follow the preferred reporting items for sysemtatic reviews and meta-analyses (PRISMA) guideline [49].

## Results

**Fig 1:**
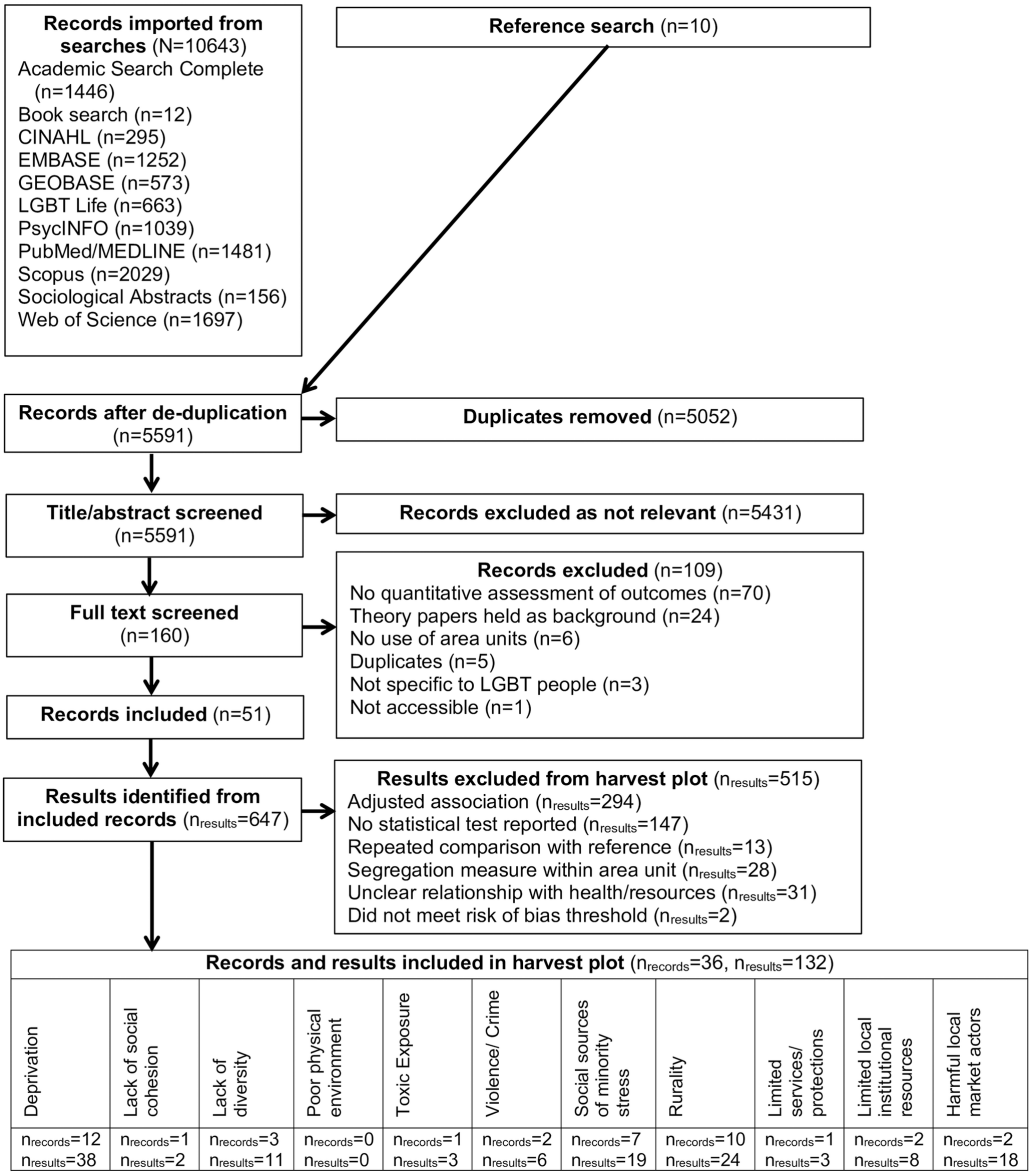
Inclusion flow diagram, Nov. 19–21, 2016.

We identified 51 records as shown in Fig 1. The mean risk of bias score was 3.37 (sd = 0.80) and ranged from 1 to 4 with 4 being the lowest risk of bias. These records contained 647 results of which 132 (from 36 records) met criteria for the harvest plot. There are distinct quantitative literatures on the spatial patterns of sexual minority lives from different disciplines ranging from HIV studies to transportation planning. The earliest identified record was published in 1985 in response to the HIV epidemic [50]. Most articles were conducted in the United States (76%), but Australia [51], China [52, 53], France [54], Germany [55], Netherlands [56], New Zealand [57], Norway [58], Sweden [31, 58, 59], and the United Kingdom [60, 61] were represented. Seven records reported on migration [29, 52, 62–66]. Five reported on segregation [29, 54, 67–69]. Only 1 record reported on sexual minority lives by racial/ethnic identity [66].

The seven records on migration included work comparing same-sex sexual behaviors of Chinese rural-to-urban migrants against rural and urban non-migrants [52], reasons for moving to or remaining in a sexual minority enclave [62], legal environment of top in- and out-migration by sexual minorities Public Use Microdata Areas (PUMAs) [63], regional characteristics associated with same-sex couple net migration [29], assessing changes in rurality over time in a study comparing risks of depression over time based on changes in neighborhood characteristics among sexual minority adolescents in the National Longitudinal Study of Adolescent Health (AddHealth) [64], rurality at age 14–16 and at the time of the survey by sexual behavior [65], and the odds of migration from the birth state by partnership status [66]. The five records on segregation identified dissimilarity indices by partnership type in the USA in 2000 [29], changes in segregation (concentration index) of gay men (operationalized as mailing addresses for a gay magazine’s subscribers) from heterosexual men in Paris over time [54], correlations between same-sex partner prevalence rates and city same-sex couple exposure indices [67], and changes over time in same-sex couple segregation in the USA [68, 69]. The one record on race/ethnicity identified odds of migration from birth state by the racial composition of partnerships [66].

Studies measured sexual orientation or a proxy thereof in a variety of ways. Twenty-six records defined sexual orientation by cohabitating same-sex partners [13, 28, 29, 32, 56, 63, 66–85]. Eleven used individual sexual orientation identity [50, 51, 53, 55, 60, 62, 64, 86–89]. Five used individual sexual behavior [52, 61, 65, 90, 91]. Two used marriage/legal partnership records [58, 92]. Two used subscriber lists or mailing lists [54, 93]. Five used multiple measures of sexual orientation, including marriage, identity, attraction, behavior, web site membership, and mailing lists [31, 57, 59, 94, 95]. We excluded two innovative studies [96, 97] using real estate listings in gay/lesbian newspapers because they did not meet our inclusion criteria.

We first report the spatial scales used in the identified records. We follow with correlates of sexual minority concentration at regional and neighborhood area units. Lastly, we report on the use of longitudinal data and racial/ethnic identity in the literature.

### Area units

Reflecting the heavy influence of U.S. records, the most common area unit was the census tract, which was used in 13 records [68, 73, 76, 77, 79–83, 89, 90, 92, 93]. The second most common area unit was the Metropolitan Statistical Area (MSA) used in 11 records [13, 28, 32, 71, 72, 74, 84, 85, 88, 91, 95]. Five records used cities or U.S. census places [31, 56, 61, 67, 69]. Four used postal codes [51, 78, 86, 94]. Four used PUMAs [63, 66, 75, 80]. Two used counties [70, 89], and one used block groups [64]. One record used a sampling grid [52]. Data zones were used in Scotland [60], arrondissements in France [54], labor market regions in Sweden [59], and one U.S. record reported using Bureau of Economic Analysis areas [29]. Other studies did not provide a clear definition of the area unit used [50, 53, 55, 57, 58, 62, 65, 87, 89].

### Correlates of spatial patterning

**Fig 2:**
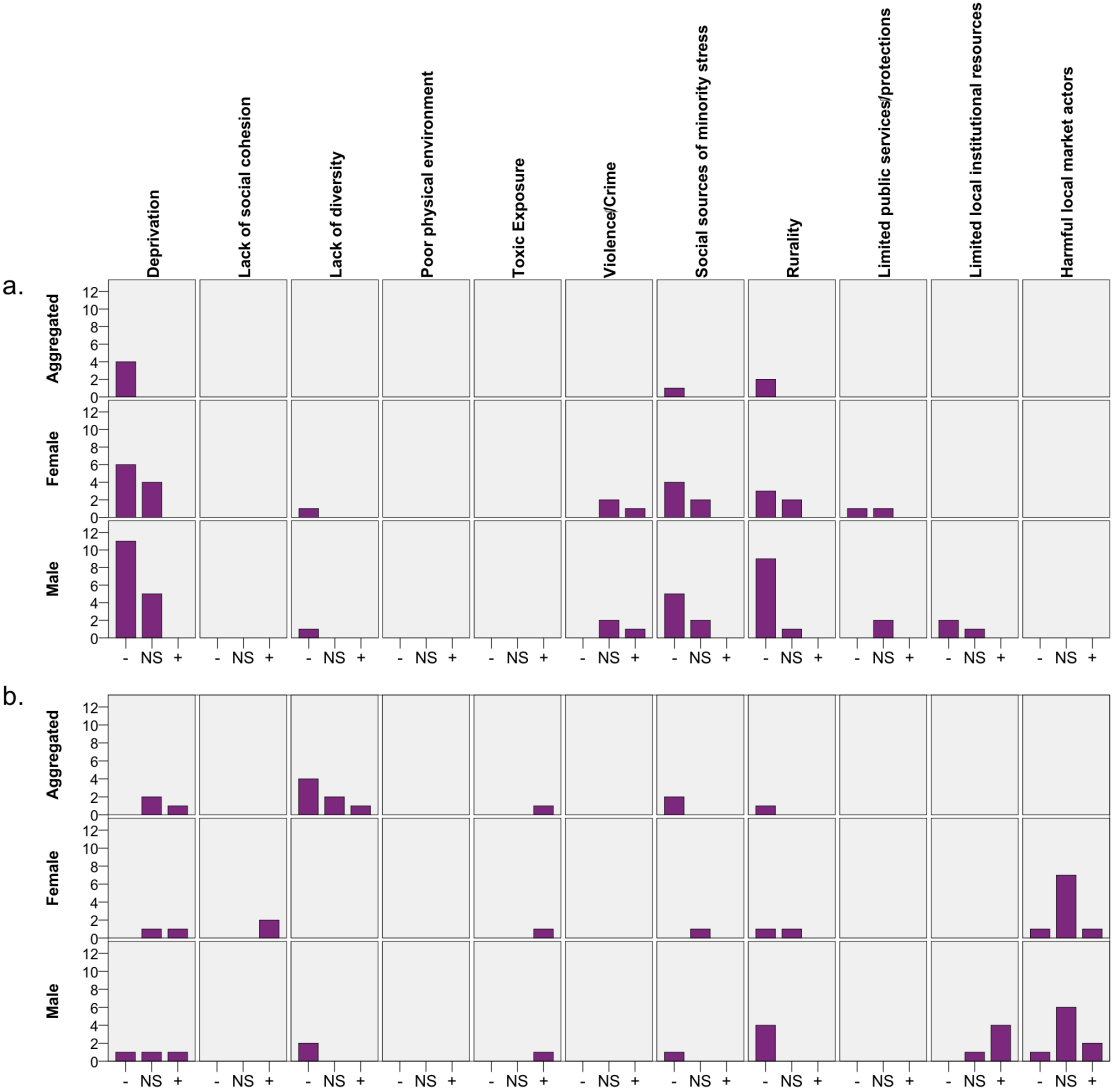
Harvest plot (count) of unadjusted relationship between sexual minority concentration and (a) regional area unit characteristics and (b) neighborhood area unit characteristics, by gender, n = 132 results from n = 36 records.

In the harvest plot (Fig 2) we show significance and direction of unadjusted associations between sexual minority concentration and area unit characteristics. Fig 2a shows these associations at larger area units (e.g., cities, metropolitan statistical areas), and Fig 2b shows these associations at smaller area units (e.g., census tracts, postal codes). The height of the bar can be read as the weight of the evidence for a negative (-), non-significant (NS), or positive association (+). In the absence of publication bias and in the absence of a “true” relationship between the variables, one would expect just 2.5% of results to fall into the negative and positive categories, respectively, when *p* was set to 0.05.

Several findings are striking: Much research has focused on deprivation and rurality. At the regional level, there is a clear pattern of findings showing sexual minority people are more likely to live in better-resourced regions and more urban regions. Similarly, sexual minority people are more likely to live in regions with more progressive values, thus potentially reducing exposure to minority stressors. At the neighborhood level, there is a clear trend towards a greater diversity of neighborhood residents and toward living in more urban neighborhoods. Given the greater resources at the regional level, it is concerning that the clear pattern of greater resources is not present in the results reported at the neighborhood level. Also concerning is that one record reported greater levels of toxic air pollution [77] and evidence suggested more limited institutional resources in neighborhoods with greater concentration of gay men [73]. There are several notable differences between men and women in the studies, mainly visible through the lack of studies of women on the neighborhood scale. A number of the domains had few or no studies.

### Longitudinal data and within-group racial/ethnic identity

Only three of the papers used longitudinal data [64, 68, 69]. One study used two waves of Add Health data to examine different county characteristics of sexual minority participants over time [64]. One used census data from two time points to assess past same-sex couple concentration with future neighborhood characteristics [68]. One used census data from two times points to associate census place characteristics with future same-sex couple concentration [69]. The remainder used cross-sectional designs, although sometimes with retrospective reporting [29, 63, 65]. Only one record reported by the racial/ethnic identity of sexual minorities [66].

## Discussion

### Principal findings

Most of our knowledge on the spatial patterning of sexual minority populations comes from the U.S.A. The available information on the spatial patterning of sexual minority individuals is clearest at larger area units (i.e., regions); there are consistent patterns indicating greater concentrations of sexual minority populations in more urban regions and in wealthier regions. At the neighborhood level, however, this pattern is not clear. Researchers have long recognized the modifiable area unit problem [98, 99]; that is, the use of different area units may result in different results that may not generalize to other area units. It is not clear to us, given the existing literature, if living in regions with more resources translates into living in better resourced neighborhoods for sexual minority people. What is clear is that there are substantial gaps in the literature regarding health-promoting or health-hindering area characteristics associated with sexual minority concentration.

Our results can be used to strengthen the existing literature. For example, work on structural stigma [100] and syndemic theory [101] is tied closely to exposure to policy and internal migration, respectively. Similarly, the commonly used minority stress model [102] suggests (but does not state directly) the importance of where people live: General and distal stressors include the role of policy and the social/political climate in which one lives. Life course and cumulative dis/advantage approaches are linked to the resources and environmental characteristics present at critical developmental stages [103, 104]. Research addressing spatial gaps in this literature could give a clearer population-level view of the role of policy and migration for studies assessing structural stigma, syndemics, and minority stress. A clearer sense of regional and neighborhood patterns across the life-course could inform research on sexual minority aging as well as adolescent development. Further work could be used to refine these theories, identify within-group resiliencies, and address gaps in the literature—particularly the limited quantitative literature about sexual minority internal migration.

Notably, our findings show a similar pattern of results by gender. Substantial theorizing has focused on the role of gender in sexual minority neighborhood formation [35]. While our focus on unadjusted, quantitative associations limits what conclusions we can draw, it may be that patterns of regional and neighborhood characteristics by gender are the same but the magnitude of the pattern differs.

### Methods

Regarding methods, it is clear that future reviews would benefit from authors and journals following reporting guidelines for observational studies that explicitly call for reporting of unadjusted results [105]. We would also suggest greater attention to the perspective that statistically holding constant other neighborhood characteristics produces a counterfactual neighborhood that does not reflect lived experience on the ground [106]. Controlling for neighborhood racial/ethnic composition, for example, may help isolate the contribution of another variable. However, real life is not held constant, and adjusted models can mask disparities that truly exist. Hand-in-hand with the limited use of longitudinal designs and despite substantial qualitative and theoretical work on migration, there is minimal assessment of migration in the identified records. We were surprised by this as longitudinal register data on legalized same-sex partnerships/marriages has been available since the late 1990s in many countries in Western Europe, and researchers could make use of this to study migration. Measurement of sexual orientation also presents a challenge. Sexual orientation is considered to have three domains, identity, behavior, and attraction [107], and same-sex partnered individuals should not be equated with LGB individuals [31]. Use of different sexual orientation domains can result in different results even in the same dataset [108]. While HIV researchers typically focus on behavior, tobacco control researchers, for example, typically focus on identity as industry marketing is targeted to LGB-identified individuals. This means that the definition of sexuality needs to be critically assessed in future studies.

### Gaps in the literature

By analyzing the different categories in line with Galster [1] and Bernard [3], we can locate several gaps in the evidence base. For example, in the Social-interactive domain, there is a lack of studies that include variables connected to social networks and family support. These are two factors that theoretical studies have argued are important for understanding the wellbeing of sexual minority populations [36, 39, 41]. Further, in the Environment domain, studies highlighting the physical environment and crime are missing. This is surprising for two reasons: (1) there is a substantial “broken windows” line of research in sociology and city planning and (2) there is a theoretical and qualitative literature on gay men as agents of gentrification [109, 110]. Crime and perceptions of crime are important in how neighborhoods are viewed and change [111]. Furthermore, the social epidemiological literature connects perceptions of neighborhoods (e.g., perceived neighborhood social cohesion) to a multitude of health outcomes; this has not been explored in the identified literature. However, one recent paper published after our search revealed that sexual minority adults report lower levels of perceptions of living in close-knit neighborhoods, of the ability to count on their neighbors, of trust of their neighbors, and of people in their neighborhoods helping each other out compared to their heterosexual counterparts [112]. In the Geographical domain, there is a gap of studies emphasizing spatial mismatch between jobs and residential location. There is evidence that sexual minority individuals sort into certain occupations and are subject to employment discrimination [113]. Future research should investigate if sexual minority people are forced to look for employment options across larger geographic scales compared to the general population. For example, how might employment-related factors be driving forces shaping migration patterns across multiple geographic scales? Finally, variables measuring stigmatization are also missing in these studies from the Institutional domain.

Beyond these domains, it is clear that very little quantitative work has examined the spatial patterning of racial/ethnic identity within LGB populations. Nor has work examined other within-group variability (e.g., immigration or socioeconomic status) in spatial patterning beyond gender and sexual orientation. The spatial patterns of bisexually-identified or transgender people are largely absent, which likely represents a limitation of many data sources to study these populations meaningfully.

### Strengths and limitations of this review

This review had a number of strengths including systematic searching developed by a professional medical librarian, implementation of the search in 11 academic databases, citation searching, dual independent coding, and an interdisciplinary team of authors. However, like all systematic reviews, it has limitations. The results are limited by biases in what is published versus what is filed away for not being “exciting enough” (i.e., publication bias). We did not conduct a search of the grey literature or solicit unpublished papers. We visually report only unadjusted results, and a substantial portion of the records did not report their unadjusted results. For example, the classic *Gay and Lesbian Atlas* reports rich quantitative data but provides no inferential statistical test results [78]. Our results come largely from the U.S.A. and may not generalize to other parts of the world. Because of the limited available literature, we opted to focus on narrow measures of risk of bias. Our risk of bias index rated records as having high quality based largely on indicators of sampling and statistical methods. This is in part due to the heavy use of census and registry data, which fared well in our risk of bias index. Future reviews should consider unique indicators of risk of bias for spatial studies. Additionally, we conducted this research from a post-positivist perspective, and it does not represent other interpretive or critical [114] ways of knowing.

### Conclusion

Even small associations can have a substantial population-level impact [115]. We know more about the regions in which sexual minority individuals live than about the characteristics of neighborhoods. We identified little quantitative evidence regarding the characteristics of neighborhoods or regions associated with sexual minority migration. Large gaps in knowledge remain. While it is promising for health that sexual minority individuals’ regional location is associated with greater resources, it is concerning—given the potential for ecological fallacy—that there is little knowledge about their location within those regions.

## Supporting information

S1 Table Evidence table. (PDF)
