# Supplementary material for: Health-related regional and neighborhood correlates of sexual minority concentration: A systematic review
Source: PLoS One. 2018 Jun 27;13(6):e0198751. doi: 10.1371/journal.pone.0198751 (PMC6021065; doi:10.1371/journal.pone.0198751)
Supplement: S1 Table — (PDF) [file pone.0198751.s001.pdf]

Health-related regional and neighborhood correlates of sexual minority concentration: A systematic review: Evidence Table

RoB *	Author 1 Year	Study Design, Year, N of individuals/couples	Sampling Frame: Sampling Strategy; definition of sexual orientation	Study area [Country: Location, Area Unit(s) of Analysis, N of area units]	Type of Quantitative Analysis Utilized	**Outcome(s)** (Bold indicates inclusion in Harvest Plot)
4	(Adler & Brenner, 1992)	Cross-sectional study, NR, 1150	Mail Addresses from two lesbian organizations in unknown U.S. city, census, membership in the mailing list	U.S., census tracts, NR (12 high lesbian concentration tracts)	*t*-test	• The percent of all females age 20-54 who are living alone was higher in lesbian than on-lesbian tracts (20.3% vs. 11.5%), p<0.001.<br>• **Median monthly contract rent was lower in lesbian than non-lesbian tracts ($201 vs. $216), p<0.01.**<br>• The percent of the total population who are female, non-family householders, not living alone (excluding women >55) was higher in the lesbian tracts than the non-lesbian tracts (2.8% vs. 1.3%), p<0.001.<br>• The percentage of all families with children <18 with a female head of household was higher in lesbian than non-lesbian tracts (31.6% vs. 24.7%), p<0.001.<br>• The percentage of households that were married families with children <18 was lower in lesbian than non-lesbian tracts (11.9% vs. 19.0%), p<0.001.<br>• **The percentage of owner-occupied housing units was lower in lesbian than non-lesbian tracts (32.0% vs. 53.4%), p<0.001.**<br>• **The percentage of people living in non-family, non-relative households (excluding women over 55 living alone) was higher in lesbian than non-lesbian tracts (32.0% vs. 19.0%), p<0.001.**
4	(Anacker & Morrow-Jones, 2005)	Cross-sectional, 2000, NR	U.S. Counties, Census, Unmarried same-sex partner households	USA: Top 50 same-sex male population counties and top 50 same-sex female couple population counties, census tracts, n=5,690 women and n=12,377 men	Poisson regression (separate analyses for each dataset of 50 counties)	• In Poisson regression adjusted for all other tract characteristics, the arts index at the metropolitan level was positively associated with the count of female same-sex couples, b=0.00, p<0.001<br>• In Poisson regression adjusted for all other tract characteristics, the arts index at the metropolitan level was positively associated with the count of same-sex male couples, b=0.2, p<0.001<br>• In Poisson regression adjusted for all other tract characteristics, the health care index at the metropolitan level was positively associated with the count of same-sex male couples, b=0.00, p<0.001<br>• In Poisson regression adjusted for all other tract characteristics, the number of new jobs at the metropolitan level was positively associated with the count of same-sex male couples, b=0.01, p<0.001<br>• In Poisson regression adjusted for all other tract characteristics, the recreation index at the metropolitan level was negatively associated with the count of same-sex male couples, b=-0.01, p<0.001<br>• In Poisson regression adjusted for all other tract characteristics, the recreation index at the metropolitan level was not associated with the count of female same-sex couples, b=-0.00, p=0.15<br>• In Poisson regression adjusted for all other tract characteristics, the tract cost of living was positively associated with the count of male same-sex couples, b=0.01, p<0.001.<br>• In Poisson regression adjusted for all other tract characteristics, the

RoB *	Author 1 Year	Study Design, Year, N of individuals/couples	Sampling Frame: Sampling Strategy; definition of sexual orientation	Study area [Country: Location, Area Unit(s) of Analysis, N of area units]	Type of Quantitative Analysis Utilized	**Outcome(s)** (Bold indicates inclusion in Harvest Plot)
						tract median household income was positively associated with the count of same-sex male couples, b=2.85^{-06}, p<0.001<br>• In Poisson regression adjusted for all other tract characteristics, the tract proportion of the population age 25 years and over with a graduate degree was positively associated with the count of same-sex male couples, b=0.24, p<0.001,<br>• In Poisson regression adjusted for all other tract characteristics, the proportion of the tract population identifying as non-Hispanic White positively was associated with the count of same-sex male couples, b=0.27, p<0.001,<br>• In Poisson regression adjusted for all other tract characteristics, the climate index at the metropolitan level was positively associated with the count of same-sex male couples, b=0.01, p<0.001<br>• In Poisson regression adjusted for all other tract characteristics, the crime index at the metropolitan level was negatively associated with the count of same-sex male couples, b=-0.00, p=0.02,<br>• In Poisson regression adjusted for all other tract characteristics, the proportion of the tract population 18 and under was negatively associated with the count of same-sex female couples, b=0.-0.29, p<0.001.<br>• In Poisson regression adjusted for all other tract characteristics, the tract home ownership rate was positively associated with the count of female same-sex couples, b=0.28, p<0.001.<br>• In Poisson regression adjusted for all other neighborhood characteristics (male model r^2=0,260, female model r^2=0,104): In Poisson regression adjusted for all other tract characteristics, the proportion of the tract population 18 and under was negatively associated with the count of same-sex male couples, b=-4.48, p<0.001,<br>• In Poisson regression adjusted for all other tract characteristics, the climate index at the metropolitan level was positively associated with the count of female same-sex couples, b=0.01, p<0.001<br>• In Poisson regression adjusted for all other tract characteristics, the crime index at the metropolitan level was positively associated with the count of female same-sex couples, b=0.01, p<0.001<br>• In Poisson regression adjusted for all other tract characteristics, the education index at the metropolitan level was negatively associated with the count of same-sex male couples, b=-0.01, p<0.001,<br>• In Poisson regression adjusted for all other tract characteristics, the education index at the metropolitan level was not significant for the count of female same-sex couples, b=-0.00, p=0.20<br>• In Poisson regression adjusted for all other tract characteristics, the

RoB*	Author 1 Year	Study Design, Year, N of individuals/couples	Sampling Frame: Sampling Strategy; definition of sexual orientation	Study area [Country: Location, Area Unit(s) of Analysis, N of area units]	Type of Quantitative Analysis Utilized	**Outcome(s)** (Bold indicates inclusion in Harvest Plot)
						health care index at the metropolitan level was negatively associated with the count of female same-sex couples, b=-0.00, p<0.001<br>• In Poisson regression adjusted for all other tract characteristics, the number of new jobs at the metropolitan level was positively associated with the count of female same-sex couples, b=0.002, p<0.001.<br>• In Poisson regression adjusted for all other tract characteristics, the tract age of housing was positively associated with the count of female same-sex couples, b=0.01, p<0.001.<br>• In Poisson regression adjusted for all other tract characteristics, the tract age of housing was positively associated with the count of same-sex male couples, b=0.01, p<0.001,<br>• In Poisson regression adjusted for all other tract characteristics, the tract cost of living was positively associated with the count of female same-sex couples, b=0.001, p=0.005.<br>• In Poisson regression adjusted for all other tract characteristics, the tract cost of living was positively associated with the count of same-sex male couples, p<0.001, and the count of female same-sex couples, p<0.001.<br>• In Poisson regression adjusted for all other tract characteristics, the tract home ownership rate was negatively associated with the count of same-sex male couples, b=-0.70, p<0.001,<br>• In Poisson regression adjusted for all other tract characteristics, the tract median household income was negatively associated with the count of female same-sex couples, b=-3.43^{-06}, p<0.001.<br>• In Poisson regression adjusted for all other tract characteristics, the tract median value of housing units was positively associated with the count of same-sex male couples, b=6.03-07, p<0.001,<br>• In Poisson regression adjusted for all other tract characteristics, the tract median value of housing units was positively the count of female same-sex couples, b=2.41-07, p<0.001.<br>• In Poisson regression adjusted for all other tract characteristics, the tract number of female same-sex couples was positively associated with the count of male same-sex couples, b=0.03, p<0.001<br>• In Poisson regression adjusted for all other tract characteristics, the tract number of male same-sex couples was positively associated with the count of female same-sex couples, b=0.01, p<0.001.<br>• In Poisson regression adjusted for all other tract characteristics, the tract proportion of the population 65 and older was negatively associated with the count of same-sex female couples, b=-1.79, p<0.001.<br>• In Poisson regression adjusted for all other tract characteristics, the

RoB*	Author 1 Year	Study Design, Year, N of individuals/couples	Sampling Frame: Sampling Strategy; definition of sexual orientation	Study area [Country: Location, Area Unit(s) of Analysis, N of area units]	Type of Quantitative Analysis Utilized	**Outcome(s)** (Bold indicates inclusion in Harvest Plot)
						tract proportion of the population 65 and older was negatively associated with the count of same-sex male couples, b=-1.64, p<0.001<br>• In Poisson regression adjusted for all other tract characteristics, the tract proportion of the population age 25 years and over with a graduate degree was positively associated with the count of female same-sex couples, b=0.90, p<0.001.<br>• In Poisson regression adjusted for all other tract characteristics, the tract proportion of the population identifying as non-Hispanic White was positively associated with the count of female same-sex couples, b=0.37, p<0.001.
3	(Andersson, Noack, Seierstad, & Weedon-Fekjær, 2006)	Cross-sectional, 1993-2001 (Norway) and 1995-2002 (Sweden), N's: Norway, 1,293 same-sex and 196,000 heterosexual; Sweden: 1,526 same-sex and 280,000 heterosexual	Partnered adults (population registry): Census, same-sex partnership/marriage	Norway and Sweden: Study Regions, 4	Descriptive	• In Norway, 62% of male partnerships and 45% of female partnerships involved a partner living in the city of Oslo. This compares to a mere 11% of the total Norwegian population living in Oslo<br>• In Sweden, 47% of male new partnerships and 36% of female partnerships involved a partner living in the Stockholm region, compared with 21% of registered heterosexual marriages
3	(Bailey, 1999)	A: Cross-sectional, 1992, 15,490 (420 LGB) B: Cross-sectional, NR, >525,000 household addresses	A: Voters: probability-based; identity (LGB) B: Mailing list addresses in LGB organizations; mailing list identification	A: USA B: USA, ZIP codes, 28,294	A: Descriptive B: Descriptive (ZIP code difference from MSA average)	• **In unadjusted correlations at the ZIP code level in Albuquerque, there was a positive correlation between gay addresses and lesbian addresses, r=0.9399, p<0.001.**<br>• In unadjusted correlations at the ZIP code level in Baltimore, there was a positive correlation between gay addresses and lesbian addresses, r=0.6861, p=NR<br>• In unadjusted correlations at the ZIP code level in Manhattan, there was a negative correlation between gay and lesbian addresses measure and African American residents, r=-0.4507, p=NR<br>• **In unadjusted correlations at the ZIP code level in New York City excluding Manhattan, there was a no correlation between gay and lesbian addresses measure and African American residents, r=NR, p>0.05**<br>• **In unadjusted correlations at the ZIP code level in Queens, there was a negative correlation between gay and lesbian addresses measure and African American residents, r=-0.3537, p=0.006**<br>• **In unadjusted correlations at the ZIP code level in Queens, there was a positive correlation between gay and lesbian addresses measure and Asian residents, r=0.4378, p<0.001**<br>• In unadjusted correlations at the ZIP code level, there was a negative correlation between gay and lesbian addresses and household size,

RoB *	Author 1 Year	Study Design, Year, N of individuals/couples	Sampling Frame: Sampling Strategy; definition of sexual orientation	Study area [Country: Location, Area Unit(s) of Analysis, N of area units]	Type of Quantitative Analysis Utilized	**Outcome(s)** (Bold indicates inclusion in Harvest Plot)
						ranging from r=-0.2212 in San Francisco to r=-0.7036 in Cincinnati, p<0.05.<br>• In unadjusted correlations at the ZIP code level, there was a negative correlation between gay and lesbian addresses and married households, ranging from r=0.46 to r=0.76. Significance not reported.<br>• Study A: For men and to a lesser extent women voters, there is a tendency of higher percentage of voters identifying as LGB as the population of the precinct sampled increases. For men: Large cities (above 500,000) 8,1; Medium Cities (250,000-500,000 [sic]) 8,3; Smaller Cities (50,000-250,000 [sic]) 3,5; Suburbs 2,5; Small Towns (5,000-10,000) 1,3; Rural Areas 2,3. For women: Large cities (above 500,000) 3,3; Medium Cities (250,000-500,000 [sic]) 8,4; Smaller Cities (50,000-250,000 [sic]) 2,0; Suburbs 2,1; Small Towns (5,000-10,000) 1,2; Rural Areas 1,5.<br>• In unadjusted correlations at the ZIP code level in Atlanta, there was a negative correlation between gay and lesbian addresses measure and African American residents, r=-0.3038, p=NR<br>• **In unadjusted correlations at the ZIP code level in Baltimore, there was a negative correlation between lesbian addresses and White residents, r=-0.3867, p=0.012**<br>• In unadjusted correlations at the ZIP code level in Chicago, there was a negative correlation between gay and lesbian addresses measure and African American residents, r=-0.3108, p=NR<br>• In unadjusted correlations at the ZIP code level in Houston, there was a negative correlation between gay and lesbian addresses measure and African American residents, r=NR, p=NR<br>• In unadjusted correlations at the ZIP code level in Los Angeles City, there was a positive correlation between gay and lesbian addresses measure and White residents, r=0.5426, p=NR<br>• In unadjusted correlations at the ZIP code level in New York City, there was a negative correlation between gay and lesbian addresses measure and African American residents, r=-0.2302, p=NR<br>• In unadjusted correlations at the ZIP code level in New York City, there was a positive correlation between gay and lesbian addresses measure and White residents, r=0.2674, p=NR<br>• In unadjusted correlations at the ZIP code level, there was a positive correlation between gay and lesbian addresses and percentage of single households, ranging from r=0.41 to r=0.72, p<0.05<br>• In unadjusted correlations at the ZIP code level, there was no positive correlation between gay and lesbian addresses measure and white residents (statistics NR)<br>• **In unadjusted correlations at the ZIP code level in Brooklyn,**

RoB*	Author 1 Year	Study Design, Year, N of individuals/couples	Sampling Frame: Sampling Strategy; definition of sexual orientation	Study area [Country: Location, Area Unit(s) of Analysis, N of area units]	Type of Quantitative Analysis Utilized	**Outcome(s)** (Bold indicates inclusion in Harvest Plot)
						**there was a positive correlation between gay addresses and lesbian addresses, r=0.8134, p<0.001**<br>• In unadjusted correlations at the ZIP code level in Dallas area, there was a negative correlation between gay and lesbian addresses measure and African American residents, r=-0.3108, p=NR<br>• In unadjusted correlations at the ZIP code level in Los Angeles City, there was a negative correlation between gay and lesbian addresses measure and African American residents, r=-0.4225, p=NR<br>• **In unadjusted correlations at the ZIP code level in New Orleans, there was a no correlation between gay and lesbian addresses measure and African American residents, r=NR, p>0.05**<br>• **In unadjusted correlations at the ZIP code level in Queens, there was a positive correlation between gay and lesbian addresses measure and Latino residents, r=0.4961, p<0.001**<br>• **Study B: The residential concentration of mailing list addresses in gay and lesbian activists/subscribers/donors with population density is more pronounced than among all addresses; the correlation between LGB mailing list concentration with urban-central city status is significant and positive, r=0.2110, versus for all addresses, r=0.1702 at the ZIP code level; the correlation between LGB mailing list concentration with urban-suburbs status is significant and negative, r=-0.0213, versus for all addresses; the correlation between LGB mailing list concentration with Nonurban status is significant and negative, r=-0.1886, versus for all addresses; the correlation between LGB mailing list concentration with rural-on farms status is significant and negative, r=-0.0863, versus for all addresses.**<br>• In unadjusted correlations at the ZIP code level in Baltimore, there was a negative correlation between gay and lesbian addresses and White residents, r=-0.4495, p=NR<br>• In unadjusted correlations at the ZIP code level in Chicago, there was a positive correlation between gay and lesbian addresses measure and American Indian residents, r=NR, p=NR<br>• **In unadjusted correlations at the ZIP code level in Los Angeles County, there was a positive correlation between gay and lesbian addresses measure and Anglo/White residents, r=0.2093, p<0.05**<br>• In unadjusted correlations at the ZIP code level in Manhattan, there was a positive correlation between gay and lesbian addresses measure and White residents, r=0.5768, p=NR<br>• **There is little to no evidence of a positive correlation between ZIP code gay and lesbian addresses and household income,**

RoB*	Author 1 Year	Study Design, Year, N of individuals/couples	Sampling Frame: Sampling Strategy; definition of sexual orientation	Study area [Country: Location, Area Unit(s) of Analysis, N of area units]	Type of Quantitative Analysis Utilized	**Outcome(s)** (Bold indicates inclusion in Harvest Plot)
						**statistics not reported**
3	(Bauermeister et al., 2015)	Cross-sectional, 2012, 328	Young Men who Have Sex with Men (age 18-29): Convenience, same-sex behavior	Detroit Metro Area, MI, USA: Census tracts, 231	Descriptive	• **"On average, neighborhoods included in the analysis were characterized as having greater socioeconomic dis- advantage than the average neighborhood in the DMA (M = 0.46, SD = 1.05)" (p. 2363).**
4	(Baumle, 2010)	Cross-sectional; 2000	5% Census Public Use Microdata: Census, unmarried same-sex households	U.S. Metropolitan statistical areas (MSA)	t-test	• Border MSA have a higher concentration of male same-sex unmarried households than non-border MSAs, 0.79% vs 0.53%, p<0.01<br>• Border MSAs have a higher concentration of female same-sex couples than non-border MSAs, 0.64% vs. 0.54%, respectively, p<0.01.
4	(Baumle, Compton, & Poston, 2009b)	Cross-sectional; 2000; NR	Census, unmarried same-sex partners	U.S. Census tracts (for the 40 most populous cities; NR	Exposure index, correlations, OLS regression	• A Pearson correlation between the city lesbian prevalence rate and city exposure of gay men to all heterosexuals index was negative, r=-0.20, p>0.10<br>• A Pearson correlation between the city population size and city exposure of gay men to all heterosexuals index was negative, r=-0.31 p<0.10<br>• A Pearson correlation between the city crime rate and city exposure of lesbian women to all heterosexuals index was negative, r=-0.03, p>0.10<br>• A Pearson correlation between the city presence of a sodomy law and city exposure of gay men to all heterosexuals index was not significant, r=0.12, p>0.10<br>• A Pearson correlation between the city poverty rate and city exposure of gay men to all heterosexuals index was positive, r=0.01, p>0.10<br>• A Pearson correlation between the city poverty rate and city exposure of lesbian women to all heterosexuals index was positive, r=0.06, p>0.10<br>• The average city gay male exposure to all married heterosexuals index (based on census tracts within each city) was 0.80 (sd=0.06)<br>• The average city lesbian women exposure to all gay men index (based on census tracts within each city) was 0.02 (sd=0.02)<br>• The average city lesbian women exposure to all unmarried heterosexuals index (based on census tracts within each city) was 0.15 (sd=0.03)<br>• A Pearson correlation between the city crime rate and city exposure of gay men to all heterosexuals index was negative, r=-0.07, p>0.10<br>• A Pearson correlation between the city gay prevalence rate and city exposure of gay men to all heterosexuals index was negative, r=-0.66, p<0.10<br>• A Pearson correlation between the city gay prevalence rate and city

RoB *	Author 1 Year	Study Design, Year, N of individuals/couples	Sampling Frame: Sampling Strategy; definition of sexual orientation	Study area [Country: Location, Area Unit(s) of Analysis, N of area units]	Type of Quantitative Analysis Utilized	**Outcome(s)** (Bold indicates inclusion in Harvest Plot)
						exposure of lesbian women to all heterosexuals index was negative, r=-0.64, p<0.10<br>• A Pearson correlation between the city lesbian prevalence rate and city exposure of lesbian women to all heterosexuals index was negative, r=-0.24, p>0.10<br>• A Pearson correlation between the city number of gay males and city exposure of gay men to all heterosexuals index was negative, r=-0.58, p<0.10<br>• A Pearson correlation between the city number of gay men and city exposure of lesbian women to all heterosexuals index was negative, r=-0.64, p<0.10<br>• A Pearson correlation between the city number of gay or lesbian people and city exposure of gay men to all heterosexuals index was negative, r=-0.52, p<0.10<br>• A Pearson correlation between the city number of gay or lesbian people and city exposure of lesbian women to all heterosexuals index was negative, r=-0.60, p<0.10<br>• A Pearson correlation between the city number of lesbians and city exposure of gay men to all heterosexuals index was negative, r=-0.42, p<0.10<br>• A Pearson correlation between the city number of lesbians and city exposure of lesbian women to all heterosexuals index was negative, r=-0.52, p<0.10<br>• A Pearson correlation between the city percent voting Republican and city exposure of gay men to all heterosexuals index was positive, r=0.40, p>0.10<br>• A Pearson correlation between the city population size and city exposure of lesbian women to all heterosexuals index was negative, r=-0.39, p<0.10<br>• The average city gay male exposure to all heterosexuals index (based on census tracts within each city) was 0.95 (sd=0.04)<br>• The average city gay male exposure to all unmarried heterosexuals index (based on census tracts within each city) was 0.15 (sd=0.03)<br>• The average city gay male exposure to lesbian women index (based on census tracts within each city) was 0.01 (sd=0.01)<br>• The average city lesbian women exposure to all heterosexuals index (based on census tracts within each city) was 0.96 (sd=0.03)<br>• The average city lesbian women exposure to all married heterosexuals index (based on census tracts within each city) was 0.82 (sd=0.05)
4	(Baumle, Compton, &	Cross sectional, 2000, NR	U.S. adults:, census, Same-sex households	U.S., Metropolitan Statistical Areas (MSA), 331 Non-	Correlation, OLS regression	• In adjusted linear regression at the MSA level, the rate of coupled gay males was not associated with the infant mortality rate, b=-18,

RoB *	Author 1 Year	Study Design, Year, N of individuals/couples	Sampling Frame: Sampling Strategy; definition of sexual orientation	Study area [Country: Location, Area Unit(s) of Analysis, N of area units]	Type of Quantitative Analysis Utilized	**Outcome(s)** (Bold indicates inclusion in Harvest Plot)
	Poston, 2009a)			metropolitan counties (NMC), 1221 (men)/1313 (women)		p>0.10<br>• At the non-metro county level, the adjusted model for coupled gay males with 8 predictor variables explains 20% of the variance, r^2=0.203<br>• In adjusted linear regression at the MSA level, the concentration rate of coupled lesbian females was positively associated with the rate of unemployment, b=0.90, p<0.10.<br>• In adjusted linear regression at the MSA level, the rate of coupled lesbian females was positively associated with the existence of sodomy laws covering only homosexual sex, b=2.93, p<0.10<br>• In adjusted linear regression at the non-metro county level, the rate of coupled lesbian females was negatively associated with county percent of residents voting Republican in a presidential election, b=-0.11, p<0.10<br>• In adjusted linear regression at the MSA level, the rate of coupled gay males was not associated with the existence of antidiscrimination laws in the public and private sector, b=-0.38, p>0.10<br>• In adjusted linear regression at the non-metro county level, the rate of coupled gay males was positively associated with designation as a rural county, b=7.79, p<0.10<br>• In adjusted linear regression at the MSA level, the concentration rate of coupled gay males was positively associated with the unemployment rate, b=0.39, p<0.10<br>• In adjusted linear regression at the MSA level, the rate of coupled gay males was negatively associated with the percent voting Republican in a presidential election, b=-0.11, p<0.10.<br>• In adjusted linear regression at the MSA level, the rate of coupled gay males was not associated with the presence of a sodomy law covering homosexual and heterosexual sex, b=1.48, p>0.10<br>• In adjusted linear regression at the MSA level, the rate of coupled gay males was positively associated with the heterosexual cohabitation rate, b=0.11, p<0.10<br>• In adjusted linear regression at the MSA level, the rate of coupled lesbian females was negatively associated with the existence of antidiscrimination laws in the public and private sector, b=-2.08, p<0.10<br>• In adjusted linear regression at the MSA level, the rate of coupled lesbian females was not associated with public antidiscrimination laws, b=0.70, p>0.10<br>• In adjusted linear regression at the MSA level, the rate of coupled lesbian females was not associated with the presence of a sodomy

RoB *	Author 1 Year	Study Design, Year, N of individuals/couples	Sampling Frame: Sampling Strategy; definition of sexual orientation	Study area [Country: Location, Area Unit(s) of Analysis, N of area units]	Type of Quantitative Analysis Utilized	**Outcome(s)** (Bold indicates inclusion in Harvest Plot)
						law covering homosexual and heterosexual sex, b=0.88, p>0.10<br>• In adjusted linear regression at the non-metro county level, the rate of coupled gay males was negatively associated with county location adjacent to a metropolitan area, b=-1.15, p<0.10<br>• In adjusted linear regression at the non-metro county level, the rate of coupled gay males was negatively associated with county percent of residents voting Republican in a presidential election, b=-0.05, p<0.10<br>• In adjusted linear regression at the non-metro county level, the rate of coupled lesbian females was positively associated with designation as a farm-dependent county, b=6.09, p<0.10<br>• In adjusted linear regression at the non-metro county level, the rate of coupled lesbian females was positively associated with designation as a mining-dependent county, b=5.45, p<0.10<br>• In adjusted linear regression at the non-metro county level, the rate of coupled lesbian females was positively associated with designation as a rural county, b=12.99, p<0.10<br>• In an adjusted model predicting the lesbian concentration rate at the MSA level using 12 ecological predictor variables, r^2=0.395<br>• The MSA concentration rate of male same-sex partners was positively correlated with the concentration rate of female same-sex partners for MSA, r=0.67,<br>• At the non-metro county level, the adjusted linear regression model for coupled lesbian females with 8 ecological predictor variables explains, 18% of the variance, r^2=0.179<br>• In adjusted linear regression at the MSA level, the concentration rate of coupled gay males was negatively associated with the poverty rate, b=-0.42, p<0.10<br>• In adjusted linear regression at the MSA level, the rate of coupled lesbian females was positively correlated with Southern Baptist rate, b=0.03, p<0.10<br>• In adjusted linear regression at the MSA level, the rate of coupled gay males was not associated with the existence of sodomy laws covering only homosexual sex, b=1.12, p>0.10<br>• In adjusted linear regression at the MSA level, the rate of coupled gay males was positively associated with logged population size, b=1.91, p<0.10<br>• In adjusted linear regression at the MSA level, the rate of coupled gay males was positively associated with the rate of Southern Baptists, b=0.1, p<0.10.<br>• In adjusted linear regression at the MSA level, the rate of coupled gay males was positively associated with the temperature index,

RoB *	Author 1 Year	Study Design, Year, N of individuals/couples	Sampling Frame: Sampling Strategy; definition of sexual orientation	Study area [Country: Location, Area Unit(s) of Analysis, N of area units]	Type of Quantitative Analysis Utilized	**Outcome(s)** (Bold indicates inclusion in Harvest Plot)
						b=22.14, p<0.10<br>• In adjusted linear regression at the MSA level, the rate of coupled lesbian females was negatively associated with percent voting for Republican in a presidential election, b=-0.09, p<0.10<br>• In adjusted linear regression at the MSA level, the rate of coupled lesbian females was *negatively* associated with the infant mortality rate, b=-65, p<0.10<br>• In adjusted linear regression at the MSA level, the rate of coupled lesbian females was negatively associated with the poverty rate, b=-0.77, p<0.10<br>• In adjusted linear regression at the MSA level, the rate of coupled lesbian females was not associated with logged population size, b=0.17, p>0.10<br>• In adjusted linear regression at the MSA level, the rate of coupled lesbian females was positively associated with the heterosexual cohabitation rate, b=0.34, p<0.10<br>• In adjusted linear regression at the MSA level, the rate of coupled lesbian females was positively associated with the temperature index, b=28.91, p<0.10<br>• In adjusted linear regression at the non-metro county level, the rate of coupled gay males was negatively associated with county percent of residents with a college education, b=-3.76, p<0.10<br>• In adjusted linear regression at the non-metro county level, the rate of coupled gay males was not associated with designation as a mining-dependent county, b=1.52, p>0.10<br>• In adjusted linear regression at the non-metro county level, the rate of coupled gay males was positively associated with designation as a farm-dependent county, b=3.20, p<0.10<br>• In adjusted linear regression at the non-metro county level, the rate of coupled gay males was positively associated with designation as a retirement county, b=7.58, p<0.10<br>• In adjusted linear regression at the non-metro county level, the rate of coupled lesbian females was negatively associated with county percent of residents with a college education, b=-4.01, p<0.10<br>• In adjusted linear regression at the non-metro county level, the rate of coupled lesbian females was not associated with county location adjacent to a metropolitan area, b=0.19, p>0.10<br>• In adjusted linear regression at the non-metro county level, the rate of coupled lesbian females was positively associated with designation as a retirement county, b=13.00, p<0.10<br>• In an adjusted model predicting the gay concentration rate at the MSA level using 12 ecological predictor variables, $r^2=0.42$

RoB*	Author 1 Year	Study Design, Year, N of individuals/couples	Sampling Frame: Sampling Strategy; definition of sexual orientation	Study area [Country: Location, Area Unit(s) of Analysis, N of area units]	Type of Quantitative Analysis Utilized	**Outcome(s)** (Bold indicates inclusion in Harvest Plot)
						• The non-metropolitan county concentration rate of male same-sex partners was positively correlated with the concentration rate of female same-sex partners, r=0.65 (unadjusted)<br>• In adjusted linear regression at the MSA level, the rate of coupled gay males was not associated with public antidiscrimination laws, b=-0.42, p>0.10
2	(Bennett, McElroy, Johnson, Munk, & Everett, 2015)	Cross-sectional, 2012, 4280	Pride festival attendees and contacts of LGBT-serving organizations: convenience; self-definition as sexual and/or gender minority (lesbian, gay, bisexual, transgender, queer, questioning, intersex)	U.S., Missouri, postal codes, NR	Descriptive	• SGM adults were more likely to report living in rural areas, 11.3% than non-SGM adults, 6.6%, p<0.001 (note: that data collection strategy could bias toward less willingness to travel to events for non-SGM adults)
4	(Bereitschaft & Cammack, 2015)	Cross-sectional, 2008-2012, NR	U.S. Households, Probability-based (American Community Survey), "% Gay households"	U.S., Chicago, IL, Census tracts, 1983	Correlation, OLS	• **The unadjusted tract concentration of gay households was negatively correlated with the square root of proximity to closest top schools, r=-0.108, p<0.01**<br>• **The unadjusted tract concentration of gay households was negatively correlated to the logged proximity to closest rail station, r=-0.139, p<0.01**<br>• **The unadjusted tract concentration of gay households was not correlated with the square root of open space, r=-0.010. p>0.05**<br>• **The unadjusted tract concentration of gay households was positively correlated to the square root of median housing values, r=0.113, p<0.01**<br>• In an adjusted model at the census tract level, the concentration of super creative workers ("creative core") was positively correlated to the concentration of gay households, b=0.104, p<0.01<br>• In an adjusted model at the census tract level, the concentration of workers in computer, science and engineering was positively correlated to the concentration of gay households, b=0.068, p<0.01<br>• **The unadjusted tract concentration of gay households was negatively correlated with the logged distance of the 5 closest consumption ("third places") places, r=-0.185, p<0.01**<br>• **The unadjusted tract concentration of gay households was positively correlated to logged population density, r=0.197, p<0.01**<br>• The unadjusted tract concentration of gay households was positively correlated with income diversity, r=0.087, p<0.01<br>• In an adjusted model at the census tract level, the concentration of creative workers was positively correlated to the concentration of gay households, b=0.082, p<0.01<br>• In an adjusted model at the census tract level, the concentration of

RoB*	Author 1 Year	Study Design, Year, N of individuals/couples	Sampling Frame: Sampling Strategy; definition of sexual orientation	Study area [Country: Location, Area Unit(s) of Analysis, N of area units]	Type of Quantitative Analysis Utilized	**Outcome(s)** (Bold indicates inclusion in Harvest Plot)
						workers in art, design and entertainment was positively correlated to the concentration of gay households, b=0.148, p<0.01<br>• In an adjusted model at the census tract level, the concentration of workers in education, library and training was positively correlated to the concentration of gay households, b=0.052, p<0.05<br>• **The unadjusted tract concentration of gay households was negatively correlated with the square root of proximity to closest college/university, r=-0.164, p<0.01**<br>• **The unadjusted tract concentration of gay households was positively correlated to the concentration of racial diversity, r=0.098, p<0.01**<br>• **The unadjusted tract concentration of gay households was positively correlated with linguistic diversity, r=0.073, p<0.01**
4	(Binson et al., 1995)	Cross-sectional, combined years of General Social Survey (GSS) and National Health and Social Life Survey (NHSLS) (GSS: 1988-1991, 1993-1994; NHSLS: 1992)	Men aged 18-49 in USA (GSS+NHSLS); same-gender sex partners (since age 18, in the past year or the past 5 years)	USA, standard metropolitan statistical areas (SMSAs)/counties/NR, NR	Chi-square	• **In GSS+NHSLS data from 1991-1994, men are most likely to report same-sex partner(s) in the last five years in more urban areas with a dose response of most likely in central cities of 12 largest SMSAs to least likely in other rural counties, chi-square tests, p<0.01**<br>• **In GSS+NHSLS data from 1988-1994, men are most likely to report same-sex partner(s) in the last year in more urban areas with a dose response of most likely in central cities of 12 largest SMSAs to least likely in other rural counties, chi-square tests, p<0.01**<br>• **In GSS+NHSLS data from 1989-1994, men are most likely to report same-sex partner(s) in the since age 18 in more urban areas with a dose response of most likely in central cities of 12 largest SMSAs to least likely in other rural counties, chi-square tests, p<0.03**
4	(Black, Gates, Sanders, & Taylor, 2002)	Cross-sectional, 1990, 9,241 male same-sex couples and 7802 female same-sex couples	Adults in U.S. census, Census, unmarried same-sex partners	U.S., MSAs (with over 700,000 population) or Primary Metropolitan Statistical Areas (PMSAs), 46	Linear regression	• In a linear model at the MSA level adjusted for fraction of city residents believing homosexual sex is always wrong, there was a positive association between gay couple concentration and a Gabriel/Rosenthal Index (an index of amenities), b=0.443 (t=2.10, n=29)<br>• In a full adjusted model at the MSA level, t he concentration of lesbians was positively associated with Median house value, b=0.007; no statistical association between the concentration of coupled lesbians and all other variables<br>• In a full adjusted model at the MSA level, the concentration of gay males was positively associated with Median house value b=0.022);no significant associations between The concentration of gay males and all other variables<br>• In a linear model at the MSA level adjusted for fraction of city

RoB*	Author 1 Year	Study Design, Year, N of individuals/couples	Sampling Frame: Sampling Strategy; definition of sexual orientation	Study area [Country: Location, Area Unit(s) of Analysis, N of area units]	Type of Quantitative Analysis Utilized	**Outcome(s)** (Bold indicates inclusion in Harvest Plot)
						residents believing homosexual sex is always wrong, there was a positive association between gay couple concentration and an Gyourko/Tracy Index (an index of amenities), b=0.447 (t=2.07, n=30)<br>• In a linear model at the MSA level adjusted for fraction of city residents believing homosexual sex is always wrong, there was a positive association between lesbian women concentration and the Gabriel/Rosenthal index (an index of amenities), b=0.155 (t=1.68, n=29)<br>• In a linear model at the MSA level adjusted for fraction of city residents believing homosexual sex is always wrong, there was a negative association between gay couple concentration and median house value, b=-0.021 (t=6.00, n=46), p<0.05<br>• In a linear model at the MSA level adjusted for fraction of city residents believing homosexual sex is always wrong, there was a positive association between gay couple concentration and an Index of Climate, b=0.554 (t=2.97, n=46)<br>• In a linear model at the MSA level adjusted for fraction of city residents believing homosexual sex is always wrong, there was a positive association between lesbian women<br>• In a linear model at the MSA level adjusted for fraction of city residents believing homosexual sex is always wrong, there was a positive association between lesbian women concentration and an Gyourko/Tracy Index (an index of amenities), b=0.103 (t=1.10, n=30)<br>• In a linear model at the MSA level adjusted for fraction of city residents believing homosexual sex is always wrong, there was a positive association between lesbian women concentration and median house value, b=0.007 (t=3.61, n=46)<br>• In a linear model at the MSA level adjusted for median house value, there was no association between gay male concentration and the fraction of individuals with negative attitudes towards homosexual sex, b=0.013 (t=0.54, n=46), p<0.05<br>• **In an unadjusted model at the MSA level, the concentration of gay males was negatively associated with the fraction of individuals with negative attitudes towards homosexual sex, b=-0.065 (t=2.32, n=46)**<br>• **In an unadjusted model at the MSA level, the concentration of lesbian women was negatively associated with the fraction of individuals with negative attitudes towards homosexual sex, b=-0.032 (t=2.68, n=46)**
4	(Chen, 2011)	Cross-sectional, 2000	Census, Census, same-sex	U.S., Metropolitan Statistical	OLS	• **In an unadjusted linear model at the MSA level, the Gay Index in**

RoB*	Author 1 Year	Study Design, Year, N of individuals/couples	Sampling Frame: Sampling Strategy; definition of sexual orientation	Study area [Country: Location, Area Unit(s) of Analysis, N of area units]	Type of Quantitative Analysis Utilized	**Outcome(s)** (Bold indicates inclusion in Harvest Plot)
		(1990 for gay pop), NR	couples "Gary Gates Gay Index:" % same-sex couple households / % of population living in same area	Areas (MSA), 46 (58 MSAs over 1 mil. minus some with missing data)		**1990 was associated with the Stay index 2000 (number of highly educated immigrants who arrived before 1990 and did not migrate during 1995–2000 per 1,000), b=1.957, p<0.001; model r^2=0.315**<br>• **In unadjusted correlation at the MSA level, the logged 1990 Gay Index was significantly associated with the 2002 GDP, r=0.45, p<0.05**<br>• **In unadjusted linear regression at the MSA level, the same-sex couple index was associated with the Bohemian Index (number of writers, artists, and professionals in entertainment industries per 1,000 population), b=4.285, p<0.001**<br>• **In unadjusted linear regression at the MSA level, there is a positive correlation between the Gay Index and income per capita, p<0.05 (effect size not reported)**<br>• In a linear model adjusted for climate at the MSA level, the Gay Index in 1990 was associated with the Bohemian index 2000 (number of writers, artists, and professionals in entertainment industries per 1,000 population), b=3.566, p<0.001; model r^2=0.503<br>• In a linear model adjusted for climate at the MSA level, the Gay Index in 1990 was associated with the Talent index 2000 (number of tertiary degree holders per 1,000 population, 25 years old and above, b=38.51, p<0.05; model r^2=0.218<br>• In a linear model at the MSA level adjusted for climate, the Gay Index in 1990 was associated with the Stay index 2000 (number of highly educated immigrants who arrived before 1990 and did not migrate during 1995–2000 per 1,000), b=1.546, p<0.001; model r^2=0.369<br>• In adjusted linear regressions at the MSA level controlling for human capital, there is a positive correlation between the Gay Index and income per capita, p<0.05 (effect size not reported).<br>• **In unadjusted correlation at the MSA level, the same-sex couple index was associated with the Talent Index, r=0.45, p<0.001.**
4	(Chen et al., 2015)	Cross-sectional, 2011-2013, N= 1718 men [rural residents = 580; rural migrants = 572; urban residents = 566]	Grid area stratified by districts (secondary: households): Random; Sexual behavior MSM (ever had sex with another male)	China: Wuhan, study-created grid, 60	Point estimate (estimate, 95% CI) using survey weights	• **Migrants (rural to urban) were significantly (p<0.05) more likely than non-migrant rural or non-migrant urban male residents to be an MSM (5.8%, 4.7-6.8 vs. 2.8%, 1.2-4.5 vs. 1.0, 0.0-2.4, respectively).**

RoB*	Author 1 Year	Study Design, Year, N of individuals/couples	Sampling Frame: Sampling Strategy; definition of sexual orientation	Study area [Country: Location, Area Unit(s) of Analysis, N of area units]	Type of Quantitative Analysis Utilized	**Outcome(s)** (Bold indicates inclusion in Harvest Plot)
4	(Christafore & Leguizamon, 2012)	Cross-sectional, 2000, NR	U.S. adults living in Ohio, Census, Unmarried same-sex partner household	U.S., Ohio Metropolitan Statistical Area (MSA), Census tract , NR	Spatial Autoregressive models(SAR). *Note* that this paper provides multiple modeling types; we report the SAR model	• In an adjusted spatial autoregressive model, the census tract concentration of female same-sex unmarried households was not associated with ln of housing prices, b=0.00. p>0.05<br>• In an adjusted spatial autoregressive model, the census tract concentration of male same-sex unmarried households was positively associated with ln of housing prices, b=0.07, p<0.01<br>• In an adjusted spatial autoregressive model, the census tract concentration of same-sex unmarried households was positively associated with ln of housing prices, b=0.04, p<0.01
4	(Christafore, Leguizamon, & Leguizamon, 2013)	Cross-sectional, 2000, NR	House sales in year 2000 and U.S. adults in 7 Ohio MSAs: Census, unmarried same-sex partner household (adjusted through removing heterosexuals using IPUMS)	U.S., Ohio MSA, Public Use Microdata Area (PUMA), 67. Census tract, 2007	OLS with robust standard errors for clustering in PUMAs or tracts (*note*: unit of analysis is housing transactions in year 2000)	• In an adjusted (including a % Black x same-sex couples household rate interaction) linear model restricted to areas within central city limits analyzed, the PUMA concentration of male same-sex unmarried households was positively associated with ln of housing prices, b=0.01323, p<0.05; model r^2=0.727<br>• In an adjusted (including a % Black x same-sex couples household rate interaction) linear model restricted to "census groups" with annual family income above $56,833, (i.e. median family income) analyzed, the PUMA concentration of female same-sex unmarried households was not associated with logged housing prices, b=0.00647, p>0.05; model r^2=0.683<br>• In an adjusted (including a % Black x same-sex couples household rate interaction) linear model restricted to census tracts where at least 22.4% of the population 25 and older hold a Bachelor's Degree (i.e. median percent with Bachelor's Degree), the PUMA concentration of same-sex unmarried households was positively associated with logged housing prices b=0.0149, p<0.01; model r^2=0.700<br>• In an adjusted (including a % Black x same-sex couples household rate interaction) linear model restricted to census tracts where at least 22.4% of the population 25 and older hold a Bachelor's Degree (i.e. median percent with Bachelor's Degree), the tract concentration of same-sex unmarried households was positively associated with logged housing prices b=0.02051, p<0.01; model r^2=0.700<br>• In an adjusted (including a % Black x same-sec couples household rate interaction) linear model, the PUMA concentration of female same-sex unmarried households was not associated with logged housing prices, b=0.01589, p>.05; model r^2=0.753<br>• In an adjusted (including a % Black x same-sex couples household rate interaction) linear model restricted to "census groups" with annual family income above $56,833, (i.e. median family income) analyzed, the PUMA concentration of male same-sex unmarried households was positively associated with ln of housing prices,

RoB*	Author 1 Year	Study Design, Year, N of individuals/couples	Sampling Frame: Sampling Strategy; definition of sexual orientation	Study area [Country: Location, Area Unit(s) of Analysis, N of area units]	Type of Quantitative Analysis Utilized	**Outcome(s)** (Bold indicates inclusion in Harvest Plot)
						b=0.025, $p<0.05$; model $r^2=0.683$<br>• In an adjusted (including a % Black x same-sex couples household rate interaction) linear model restricted to “census groups” with annual family income above \$56,833, (i.e. median family income) analyzed, the tract concentration of same-sex unmarried households was positively associated with ln of housing prices, b=0.02459, $p<0.01$; model $r^2=0.683$<br>• In an adjusted (including a % Black x same-sex couples household rate interaction) linear model, the MSA concentration of same-sex unmarried households was positively associated with ln of housing prices (using MSA dummies), b=0.14047, $p<0.01$; model $r^2=0.5799$)<br>• In an adjusted (including a % Black x same-sec couples household rate interaction) linear model, the PUMA concentration of male same-sex unmarried households was not associated with logged housing prices, b=0.00557, p>.05; model $r^2=0.753$<br>• In an adjusted (including a % Black x same-sec couples household rate interaction) linear model, the tract concentration of same-sex unmarried households was positively associated with logged housing prices, b=02230, $p<0.01$; model r^2=0,75<br>• In an adjusted (including a % Black x same-sec couples household rate interaction) linear model, the PUMA concentration of same-sex unmarried households was positively associated with logged housing prices, b=0.0101, $p<0.1$; model $r^2=0.752$<br>• In an adjusted (including a % Black x same-sex couples household rate interaction) linear model restricted to areas within central city limits analyzed, the PUMA concentration of same-sex unmarried households was positively associated with ln of housing prices, b=0.0140. $p<0.01$; model $r^2=0.726$<br>• In an adjusted (including a % Black x same-sex couples household rate interaction) linear model restricted to areas within central city limits analyzed, the tract concentration of same-sex unmarried households was not associated with logged housing prices, b=0.0247, $p>0.01$; model $r^2=0.7296$<br>• In an adjusted (including a % Black x same-sex couples household rate interaction) linear model restricted to census tracts where at least 22.4% of the population 25 and older hold a Bachelor's Degree (i.e. median percent with Bachelor's Degree), the PUMA concentration of male same-sex unmarried households was positively associated with logged housing prices b=0.02245, $p<0.05$; model $r^2=0.700$<br>• In an adjusted (including a % Black x same-sex couples household rate interaction) linear model restricted to “census groups” with

RoB *	Author 1 Year	Study Design, Year, N of individuals/couples	Sampling Frame: Sampling Strategy; definition of sexual orientation	Study area [Country: Location, Area Unit(s) of Analysis, N of area units]	Type of Quantitative Analysis Utilized	**Outcome(s)** (Bold indicates inclusion in Harvest Plot)
						annual family income above $56,833, (i.e. median family income) analyzed, the PUMA concentration of same-sex unmarried households was positively associated with ln of housing prices, b=0.0136, $p<0.05$; model $r^2=0.683$<br>• In an adjusted (including a % Black x same-sex couples household rate interaction) linear model restricted to areas within central city limits analyzed, the PUMA concentration of female same-sex unmarried households was not associated with logged housing prices, b=0.00686, $p>0.05$; model $r^2=0.727$<br>• In an adjusted (including a % Black x same-sex couples household rate interaction) linear model restricted to census tracts where at least 22.4% of the population 25 and older hold a Bachelor's Degree (i.e. median percent with Bachelor's Degree), the PUMA concentration of female same-sex unmarried households was not associated with logged housing prices b=0.00495, $p>0.05$; model $r^2=0.700$
4	(Collins, Grineski, & Morales, 2016)	Cross-sectional, 2011 (air pollution), 2010 (census), 2008-2010 ACS, NR	Adults: Census; same-sex coupled households	Houston, TX, USA: Census tracts, 1,023	Correlation, GEE, Descriptive	• **Same-sex male partner enclave was positively associated with indicator of cumulative cancer risk from air quality, $r_s=0.201$, $p<0.001$**<br>• In GEE analysis predicting hazardous air pollutant cancer risk and adjusting for same-sex female enclave status, population density, proportion Black, proportion Hispanic, median household income, median household income squared, Gini index, and proportion renter occupied housing, same-sex male partner enclave status was positively associated the outcome variable, b=0.114, $p<0.001$, 95% CI: 0.070-0.159<br>• **Same-sex partner enclave was positively associated with indicator of cumulative cancer risk from air quality, $r_s=0.207$, $p<0.001$**<br>• In GEE analysis predicting hazardous air pollutant cancer risk and adjusting for population density, proportion Black, proportion Hispanic, median household income, median household income squared, Gini index, and proportion renter occupied housing, same-sex partner enclave status was positively associated the outcome variable, b=0.105, $p<0.001$, 95% CI: 0.078-0.131<br>• **Same-sex female partner enclave was positively associated with indicator of cumulative cancer risk from air quality, $r_s=0.114$, $p<0.001$**<br>• In GEE analysis predicting hazardous air pollutant cancer risk and adjusting for same-sex male enclave status, population density, proportion Black, proportion Hispanic, median household income, median household income squared, Gini index, and proportion renter

RoB*	Author 1 Year	Study Design, Year, N of individuals/couples	Sampling Frame: Sampling Strategy; definition of sexual orientation	Study area [Country: Location, Area Unit(s) of Analysis, N of area units]	Type of Quantitative Analysis Utilized	**Outcome(s)** (Bold indicates inclusion in Harvest Plot)
						occupied housing, same-sex female partner enclave status was not associated the outcome variable, b=-0.028, p>0.05, 95% CI: -0.068-0.012
2	(Compton & Baumle, 2012)	Cross-Sectional, NR, 40	Convenience: Snowball; gay or lesbian	San Francisco Bay (3 counties)	Descriptive	• Reasons for moving to or remaining in enclave, % endorsed: 38% weather, 50% nature, 100% community, 73% dating, political climate 98%, legislation 55%, institutions 85%
4	(Cooke & Rapino, 2007)	Cross-sectional (retrospective report of 1995 residence) 2000, NR	All in Census, International Public Use Microdata Samples (IPUMS) version of the 5 percent PUMS of the<br>Census, Unmarried same-sex partners	U.S., Bureau of Economic Analysis areas (BEA Area); 177	Linear regressions	• In an adjusted linear model (adjusting for % urban and amenity score) at the BEA area unit, the percentage of the total U.S. population in 1995 was negatively associated with the gay couple net migration, b=-0.07, p<0.001<br>• In an adjusted linear model (adjusting for % total U.S. population lesbian couple population) at the BEA area unit, the percent of total U.S. population in 1995 was negatively associated with the lesbian couple net migration, b=-0.15, p<0.001<br>• In an adjusted linear model at the BEA area unit, the percentage of partnered lesbian couples in 1995 was positively associated with the lesbian couple net migration, b=-0.12, p<0.001<br>• In an adjusted linear model at the BEA area unit, the percentage of population with higher education attainment was not associated with the gay couple net migration, b=-0.00, p=0.49<br>• In an adjusted linear model at the BEA area unit, the percentage of total U.S. population in 1995 was not associated with the gay couple net migration, b=-0.03, p=0.41<br>• National index of dissimilarity for lesbian partners from gay partners at BEA area unit in year 2000, 16.3<br>• In an adjusted linear model at the BEA area unit, the percentage of total U.S. partnered lesbian population in 1995 was marginally associated with the lesbian couple net migration, b=-1.09, p=0.09<br>• In an adjusted linear model at the BEA area unit, the percentage of total U.S. population in 1995 was negatively associated with the lesbian couple net migration, b=-0.18, p<0.001<br>• In an adjusted linear model at the BEA area unit, the tolerance score (based on state laws) was not associated with the gay couple net migration, b=-0.00, p=0.23<br>• National index of dissimilarity for lesbian partners from total population at BEA area unit in year 2000, 17.1<br>• In an adjusted linear model (adjusting for % total U.S. population and amenity score) at the BEA area unit, the percent urban was positively associated with the gay couple net migration, b=0.002, p=0.05<br>• In an adjusted linear model (adjusting for % total U.S. population) at

RoB*	Author 1 Year	Study Design, Year, N of individuals/couples	Sampling Frame: Sampling Strategy; definition of sexual orientation	Study area [Country: Location, Area Unit(s) of Analysis, N of area units]	Type of Quantitative Analysis Utilized	**Outcome(s)** (Bold indicates inclusion in Harvest Plot)
						the BEA area unit, the percentage of the total U.S. partnered lesbian population in 1995 was positively associated with the lesbian couple net migration, b=0.09, p<0.01<br>• In an adjusted linear model at the BEA area unit, the natural amenity score was positively associated with the gay couple net migration, b=0.01, p=0.01<br>• In an adjusted linear model at the BEA area unit, the natural amenity score was positively associated with the lesbian couple net migration, b=0.00, p=0.94<br>• In an adjusted linear model at the BEA area unit, the percentage of urban was marginally associated with the gay couple net migration, b=0.002, p=0.07<br>• In an adjusted linear model at the BEA area unit, the percentage of urban was not associated with the lesbian couple net migration, b=0.001, p=0.25<br>• National index of dissimilarity for gay partners from heterosexual partners at BEA area unit in year 2000, 20.7<br>• National index of dissimilarity for lesbian partners from heterosexual partners at BEA area unit in year 2000, 15.8<br>• In an adjusted linear model (adjusting for % urban and % total U.S. population) at the BEA area unit, the percentage of the amenity score was positively associated with the gay couple net migration, b=0.01, p=0.01<br>• In an adjusted linear model at the BEA area unit, the percentage of partnered gay couples in 1995 was not associated with the gay couple net migration, b=-0.03, p=0.39<br>• In an adjusted linear model at the BEA area unit, the percentage of population with higher education attainment was not associated with the lesbian couple net migration, b=-0.00, p=0.55<br>• In an adjusted linear model at the BEA area unit, the percentage of total U.S. partnered gay population in 1995 was not associated with the gay couple net migration, b=0.64, p=0.35<br>• In an adjusted linear model at the BEA area unit, the tolerance score (based on state laws) was not associated with the lesbian couple net migration, b=0.00, p=0.70<br>• National index of dissimilarity for gay partners from total population at BEA area unit in year 2000, 19.6

RoB*	Author 1 Year	Study Design, Year, N of individuals/couples	Sampling Frame: Sampling Strategy; definition of sexual orientation	Study area [Country: Location, Area Unit(s) of Analysis, N of area units]	Type of Quantitative Analysis Utilized	**Outcome(s)** (Bold indicates inclusion in Harvest Plot)
4	(Cooke, 2005)	Cross-sectional (retrospective report of 1985 residence), 1990, N=1118	All in Census, Census, Same-sex unmarried partner cohabitation	USA: PUMA (Public Use Microdata Areas), NR	Descriptive	• Top five net in-migration PUMAS ranked 8; 24; 17; 6 and 40 on the Gay/lesbian supportive laws and 3; 14; 9; 8 and 11 on Gay/Lesbian concentration<br>• Top five net outmigration PUMAS ranked 20; 20; 12; 5 and 2 on the Gay/lesbian supportive laws and 48; 31; 44; 15 and 21 on Gay/Lesbian concentration
3	(Croes, 1996)	Cross-sectional, 1995, 25.500 men and 13.200 women	All in population register, Total, same-sex cohabiting partners with an age difference less than 20 years	Netherlands, cities, NR	Descriptive	• Extremely urbanized areas 6,8 same-sex individuals per 1 000 (men 9,5; women 4,3)<br>• Highly urbanized areas 3,3 same-sex individuals per 1 000 (men 4,3; women 2,3)<br>• Moderately urbanized areas 1,6 same-sex individuals per 1 000 (men 2,2; women 1,1)<br>• Not urbanized areas 0.2 same-sex individuals per 1 000 (men 0.3; women 0.2)<br>• Slightly urbanized areas 0.6 same-sex individuals per 1 000 (men 0.8; women 0.5)
2	(Doan & Higgins, 2009)	Cross-Sectional, NR, N=127	Tallahassee, FL, LGBT organizations: Snowball: queer identified respondents	Tallahassee, FL, NR	Descriptive (bivariate associations)	• 31.4% of gay men lived in suburban developments compared with 34.2% of lesbian women.<br>• 13.7% of gay men lived in the rural fringe compared with 30.3% of lesbian women.<br>• 54.9% of gay men lived in town vs. 35.5% of lesbian women.
3	(Elder, Rothblum, & Solomon, 2010)	Cross-sectional, July 1, 2000, to June 30, 2001, 2,475 civil union certificates from the period	Vermont Civil Union marriage licenses, same-sex civil unions	USA: ZIP codes/tracts, NR	Regression	• % Multiracial: National average (2%) vs civil unions (4%)<br>• % Asian: national average (3%) vs civil unions (8%)<br>• % Hispanic: national average (11%) vs. civil unions (7.9%)<br>• % Black: national average (13.7%) vs. civil unions (8%)<br>• Average family size: national average (3.1) vs. civil unions (3.0)<br>• Average household size: national average (2.6) vs. civil unions (2.5)<br>• Married households with children: national average (23.3) vs. civil unions (21.5)<br>• Married households without children: national average (27.7) vs. civil unions (26.6)<br>• No significant differences found between same-sex civil union census tract characteristics and average for population in regression models.<br>• Ratio of rentals to home ownership: national average (31.3:60) vs. civil unions (35.1:57.2)<br>• Sex ratios: national average 49.1:50.9 vs civil union (49.1:50.9)
4	(Ernst & Houts, 1985)	Cross-sectional, 1982, 365 gay/lesbians in PA	Gays and lesbians in Pennsylvania Organizations/bars/papers, convenience; Self-identification	Pennsylvania, N/A, NR	Descriptive	• Distribution of gay males: 52.9% (urban), 6.1% (suburban), 41.0 (rural)<br>• Distribution of gay males: 52.9% (urban), 6.1% (suburban), 41.0 (rural)

RoB *	Author 1 Year	Study Design, Year, N of individuals/couples	Sampling Frame: Sampling Strategy; definition of sexual orientation	Study area [Country: Location, Area Unit(s) of Analysis, N of area units]	Type of Quantitative Analysis Utilized	**Outcome(s)** (Bold indicates inclusion in Harvest Plot)
3	(Everett, 2014)	Longitudinal, 1994 , 1994, Wave I (N = 1,328 @ Wave III)	US adolescents in Longitudinal National Longitudinal Study of Adolescent Health (AddHealth); respondents who reported a mostly heterosexual, bisexual, mostly gay, or exclusively gay identity at Wave III	U.S.; block groups for neighborhood proxies	OLS & Logistic	• 38.6 lived in neighborhoods with 1% same-sex couples<br>• 8.35% lived in neighborhoods with between 2 and 8% same-sex couples<br>• Travel mode choice among same-sex couples<br>• 53% lived in neighborhoods with 0% same-sex couples<br>• change in % urban from Wave I to Wave III is -15%, 59.1% percent county % of voters voting Republican in 1995 and 2001 gubernatorial elections<br>• change in Republican voting between waves was 5.8%<br>• percent of people over 25 with college degrees 30.3%
4	(Fanning & Ruther, 2015)	Repeated cross-sectional, 2000 & 2010, NR	U.S. adults, Census, unmarried same-sex partners (adjusted to the tract level)	U.S., 38 large cities located in 35 MSAs, Census tracts, 10.450. *Note*: the authors report additional subgroup analyses by U.S. region.	Spatial regression	• In a spatial regression model adjusted for physical, social, and neighborhood composition tract characteristics, the concentration of gay couples in 2009 was positively associated with proportion of the city's total households in 2000. b=0.2822, z=2.89<br>• Duncan indices of dissimilarity at city level (calculated from tracts) meaningfully large across 38 cities and almost all decrease between 2000 and 2010<br>• In a spatial regression model adjusted for physical, social, and neighborhood composition tract characteristics, the concentration of lesbian couples in 2009 (DV) was not associated with % vacant housing in 2000. b=-0.0020. z=-1.21<br>• In a spatial regression model adjusted for physical, social, and neighborhood composition tract characteristics, the tract concentration of gay couples in 2009 (DV) was not associated with a measure of squared distance from the city center, b=-0.0122, z=-0.38<br>• In a spatial regression model adjusted for physical, social, and neighborhood composition tract characteristics, the concentration of gay couples in 2009 was not associated with the proportion of non-family households in 2000. b=0.0066, z=0.11<br>• In a spatial regression model adjusted for physical, social, and neighborhood composition tract characteristics, the concentration of gay couples in 2009 was positively associated with the tract ratio of median income/city median income in 2000. b=0.0006, z=3.34<br>• In a spatial regression model adjusted for physical, social, and neighborhood composition tract characteristics, the concentration of lesbian couples in 2009 (DV) was not associated with the 2000 % of housing built before 1939, b=0.0010, z=1.69<br>• In a spatial regression model adjusted for physical, social, and neighborhood composition tract characteristics, the concentration of lesbian couples in 2009 (DV) was positively associated with proportion of the city's total households in 2000. b=0.3706, z=3.14

RoB*	Author 1 Year	Study Design, Year, N of individuals/couples	Sampling Frame: Sampling Strategy; definition of sexual orientation	Study area [Country: Location, Area Unit(s) of Analysis, N of area units]	Type of Quantitative Analysis Utilized	**Outcome(s)** (Bold indicates inclusion in Harvest Plot)
						• In a spatial regression model adjusted for physical, social, and neighborhood composition tract characteristics, the concentration of lesbian couples in 2009 (DV) was positively associated with the concentration of lesbian couples in 2000. b=0.3718, z=14.68<br>• In a spatial regression model adjusted for physical, social, and neighborhood composition tract characteristics, the concentration of lesbian couples in 2009 (DV) was not associated with the proportion of non-family households in 2000. b=-0.0875, z=-1.20<br>• In a spatial regression model adjusted for physical, social, and neighborhood composition tract characteristics, the concentration of lesbian couples in 2009 (DV) was not associated with the tract ratio of median income/city median income in 2000. b=0.0001, z=0.65<br>• In a spatial regression model adjusted for physical, social, and neighborhood composition tract characteristics, the concentration of lesbian couples in 2009 (DV) was not associated with tract proportion of the city's total Black population in 2000. b=-0.0312, z=-1.61<br>• In a spatial regression model adjusted for physical, social, and neighborhood composition tract characteristics, the concentration of lesbian couples in 2009 (DV) was not associated with tract proportion of the city's total Hispanic population in 2000. b=-0.0345, z=-1.93<br>• In a spatial regression model adjusted for physical, social, and neighborhood composition tract characteristics, the concentration of lesbian couples in 2009 (DV) was positively associated with the concentration of gay couples in 2000. b=0.1668, z=8.86<br>• In a spatial regression model adjusted for physical, social, and neighborhood composition tract characteristics, the tract income growth from 2000 to 2009 (DV), was positively associated with the concentration of gay couples in 2000, b=2.8233, z=6.08<br>• In a spatial regression model adjusted for physical, social, and neighborhood composition tract characteristics, the tract population growth from 2000 to 2009 (DV), was not associated with the concentration of lesbian couples in 2000, b=-0.0025, z=-0.86<br>• In a spatial regression model adjusted for physical, social, and neighborhood composition tract characteristics, the tract population growth from 2000 to 2009 (DV), was positively associated with the concentration of gay couples in 2000. b=0.0055, z=2.50<br>• In a spatial regression model adjusted for physical, social, and neighborhood composition tract characteristics, the concentration of gay couples in 2009 (DV) was not associated with a measure of distance from the city center, b=0.0007, z=0.11

RoB*	Author 1 Year	Study Design, Year, N of individuals/couples	Sampling Frame: Sampling Strategy; definition of sexual orientation	Study area [Country: Location, Area Unit(s) of Analysis, N of area units]	Type of Quantitative Analysis Utilized	**Outcome(s)** (Bold indicates inclusion in Harvest Plot)
						• In a spatial regression model adjusted for physical, social, and neighborhood composition tract characteristics, the concentration of gay couples in 2009 (DV) was not associated with the 2000 % of housing built before 1939, b=0.0002, z=0.41<br>• In a spatial regression model adjusted for physical, social, and neighborhood composition tract characteristics, the concentration of gay couples in 2009 (DV) was not associated with the 2000 population density (100,000), b=0.0002, z=0.35<br>• In a spatial regression model adjusted for physical, social, and neighborhood composition tract characteristics, the concentration of gay couples in 2009 (DV) was positively associated with 2000 city proportion of detached single family homes, b=0.0719, z=2.20<br>• In a spatial regression model adjusted for physical, social, and neighborhood composition tract characteristics, the concentration of gay couples in 2009 was negatively associated with tract proportion of the city's total Black population in 2000. b=-0.0754, z=-4.68<br>• In a spatial regression model adjusted for physical, social, and neighborhood composition tract characteristics, the concentration of gay couples in 2009 was not associated with the concentration of lesbian couples in 2000. b=0.0237, z=1.13<br>• In a spatial regression model adjusted for physical, social, and neighborhood composition tract characteristics, the concentration of gay couples in 2009 was not associated with tract proportion of the city's total Hispanic population in 2000. b=-0.0119, z=-0.81<br>• In a spatial regression model adjusted for physical, social, and neighborhood composition tract characteristics, the concentration of gay couples in 2009 was positively associated with % vacant housing in 2000. b=0.0029, z=2.13<br>• In a spatial regression model adjusted for physical, social, and neighborhood composition tract characteristics, the concentration of gay couples in 2009 was positively associated with the concentration of gay couples in 2000. b=0.7957, z=51.06<br>• In a spatial regression model adjusted for physical, social, and neighborhood composition tract characteristics, the concentration of lesbian couples in 2009 (DV) was not associated with a measure of distance from the city center, b=0.0053, z=0.68<br>• In a spatial regression model adjusted for physical, social, and neighborhood composition tract characteristics, the concentration of lesbian couples in 2009 (DV) was not associated with the 2000 population density (100,000), b=0.00, z=-0.06<br>• In a spatial regression model adjusted for physical, social, and neighborhood composition tract characteristics, the concentration of

RoB*	Author 1 Year	Study Design, Year, N of individuals/couples	Sampling Frame: Sampling Strategy; definition of sexual orientation	Study area [Country: Location, Area Unit(s) of Analysis, N of area units]	Type of Quantitative Analysis Utilized	**Outcome(s)** (Bold indicates inclusion in Harvest Plot)
						lesbian couples in 2009 (DV) was positively associated with 2000 city proportion of detached single family homes, b=0.1761, z=4.46<br>• In a spatial regression model adjusted for physical, social, and neighborhood composition tract characteristics, the tract concentration of lesbian couples in 2009 (DV) was not associated with a measure of squared distance from the city center, b=-0.0244, z=-0.63<br>• In a spatial regression model adjusted for physical, social, and neighborhood composition tract characteristics, the tract income growth from 2000 to 2009 (DV), was not associated with the concentration of lesbian couples in 2000, b=0.4709, z=0.75<br>• Moran's I for both lesbian and gay same-sex couples across 38 cities, almost all significant with larger clustering for gay couples.
3	(Farmer, Blosnich, Jabson, & Matthews, 2016)	Cross-sectional, 2010, 89,677 (1952 LGB adults)	Adults with phones: probability based: identity	10 U.S. states (Alaska, Arizona, California, Maine, Massachusetts, Montana, New Mexico, North Dakota, Washington, and Wisconsin), MSA, NR	Descriptive	• Among men living in rural (i.e., non MSA) areas, 0.94% identified as gay, 0.64% identified as bisexual, and 98.42% identified as heterosexual compared to among non-rural men for whom 2.33% identified as gay, 0.70% identified as bisexual, and 96.97% identified as straight.<br>• Among women who identified as rural (i.e., outside of MSA), 0.78% identified as lesbian, 0.90% identified as bisexual, and 98.31% identified as straight compared to non-rural women for whom 1.32% identified as lesbian, 0.84% identified as bisexual, and 97.84% identified as straight.
4	(Fasula et al., 2016)	Cross-sectional, 2002+2006-2010+2011-2013, 8,068	Males age 15-24 in USA; probability-based (National Survey on Family Growth), attraction or identity	USA, MSA, NR	Logistic regression	• **Young sexual minority males were significantly less likely to live in other metropolitan areas than in the central city, PR=0.7, 95% CI: 0.6-0.9, p<0.05**<br>• **Young sexual minority males were not significantly less likely to live in non-metropolitan areas than in the central city, PR=0.9, 95% CI: 0.8-1.1, p>0.05**
4	(Florida, 2002)	Cross-sectional: Census, 1990, 50 largest MSAs (individuals/couples NR)	Census derived same-sex households ("The index is basically a location quotient that measures the number of gay households compared to the national population of gay households divided by the	U.S. MSAs	Pearson Correlations, Regression	• **In unadjusted models, the gay index is associated with an index of Coolness, r=0.3769, p<0.05**<br>• **In unadjusted models, the gay index is associated with income, r=0.4983, p<0.01**<br>• **In unadjusted models, the gay index is not associated with change in income (from 1991 to 1997), r=0.1991, p>0.10**<br>• **In unadjusted models, the gay index is associated with a Culture index, r=0.2886, p<0.10**<br>• In adjusted models, the gay index ("diversity index") is positively correlated with Tech-Pole index, p=0.0001<br>• In unadjusted models the gay index is associated with the better Climate, r=0.4466, p<0.01

RoB	Author 1 Year	Study Design, Year, N of individuals/couples	Sampling Frame: Sampling Strategy; definition of sexual orientation	Study area [Country: Location, Area Unit(s) of Analysis, N of area units]	Type of Quantitative Analysis Utilized	**Outcome(s)** (Bold indicates inclusion in Harvest Plot)
						• **In unadjusted models, the gay index is associated with the Talent Index, r=0.7181, p<0.01**<br>• In adjusted models, the gay index ("diversity index") is positively correlated with talent availability, p=0.0001<br>• **In unadjusted models, the gay index is associated with the Tech index, r=0.7677, p<0.01**<br>• **In unadjusted models, the gay index is not associated with a Recreation index, r=0.1568, p>0.10**<br>• **In unadjusted models, the gay index is not associated with median house value, r=0.4464, p>0.10**
3	(Gates & Ost, 2004)	Cross-sectional, 2000, NR	USA: Census, same-sex couples	USA, postal codes	Descriptive	• At the county level, characteristics of female same-sex couples show higher levels of crime than married opposite-sex couples.<br>• At the county level, characteristics of male same-sex couples show higher levels of crime than unmarried opposite-sex couples.<br>• The typical ZIP code characteristics of female same-sex couples show a greater percentage of gay male couples than in typical married opposite-sex couple neighborhoods.<br>• The typical ZIP code characteristics of female same-sex couples show a greater percentage of gay male couples than in typical unmarried opposite-sex couple neighborhoods.<br>• The typical ZIP code characteristics of female same-sex couples show lower share of homes with three or more bedrooms than for married opposite-sex couples.<br>• The typical ZIP code characteristics of female same-sex couples show no difference in the share of homes with three or more bedrooms than for unmarried opposite-sex couples.<br>• The typical ZIP code characteristics of male same-sex couples show a greater percentage of gay male couples than in typical unmarried opposite-sex couple neighborhoods.<br>• The typical ZIP code characteristics of male same-sex couples show lower share of homes with three or more bedrooms than for married opposite-sex couples.<br>• The typical ZIP code characteristics of male same-sex couples show lower share of homes with three or more bedrooms than for unmarried opposite-sex couples.<br>• At the county level, characteristics of female same-sex couples show higher levels of crime than unmarried opposite-sex couples.<br>• The typical ZIP code characteristics of female same-sex couples show higher rates of non-English speaking residents than for opposite-sex couples. (p. 36)<br>• The typical ZIP code characteristics of female same-sex couples show higher share of homes built before 1939 than for married

RoB*	Author 1 Year	Study Design, Year, N of individuals/couples	Sampling Frame: Sampling Strategy; definition of sexual orientation	Study area [Country: Location, Area Unit(s) of Analysis, N of area units]	Type of Quantitative Analysis Utilized	**Outcome(s)** (Bold indicates inclusion in Harvest Plot)
						opposite-sex couples.<br>• The typical ZIP code characteristics of female same-sex couples show a higher percentage of unmarried couples than in typical married opposite-sex couple neighborhoods.<br>• The typical ZIP code characteristics of female same-sex couples show a lower percentage of married couples than in typical married opposite-sex couple neighborhoods.<br>• The typical ZIP code characteristics of female same-sex couples show higher median individual income than for unmarried opposite-sex couples. (p. 36)<br>• The typical ZIP code characteristics of female same-sex couples show higher rates of college graduates than for opposite-sex couples. (p. 36)<br>• The typical ZIP code characteristics of female same-sex couples show higher rates of foreign born residents than for opposite-sex couples. (p. 36)<br>• The typical ZIP code characteristics of female same-sex couples show higher rates of non-white residents than for opposite-sex couples. (p. 36)<br>• The typical ZIP code characteristics of female same-sex couples show no difference in the percentage of married couples than in typical unmarried opposite-sex couple neighborhoods.<br>• The typical ZIP code characteristics of male same-sex couples show a higher percentage of unmarried couples than in typical married opposite-sex couple neighborhoods.<br>• The typical ZIP code characteristics of male same-sex couples show a lower percentage of married couples than in typical married opposite-sex couple neighborhoods.<br>• "Same-sex couples have the highest probability of living in an urban area among the different couple types. A gay-male couple's neighborhood is more likely to be urban than that of a lesbian couple's, and both are more likely to be urban than the neighborhoods of either a heterosexual unmarried partner or a married couple" (p. 35).<br>• At the county level, characteristics of female same-sex couples show higher levels of crime than opposite-sex couples.<br>• At the county level, characteristics of male same-sex couples show higher levels of crime than married opposite-sex couples.<br>• At the county level, characteristics of male same-sex couples show higher levels of crime than opposite-sex couples.<br>• The typical typical ZIP code characteristics of male same-sex couples show higher rates of non-white residents than for opposite-

RoB*	Author 1 Year	Study Design, Year, N of individuals/couples	Sampling Frame: Sampling Strategy; definition of sexual orientation	Study area [Country: Location, Area Unit(s) of Analysis, N of area units]	Type of Quantitative Analysis Utilized	**Outcome(s)** (Bold indicates inclusion in Harvest Plot)
						sex couples. (p. 36)<br>• The typical ZIP code characteristics of female same-sex couples show a lower percentage of unmarried couples than in typical unmarried opposite-sex couple neighborhoods.<br>• The typical ZIP code characteristics of female same-sex couples show no difference in median individual income than from married opposite-sex couples. (p. 36)<br>• The typical ZIP code characteristics of female same-sex couples show no difference in the share of homes built before 1939 compared to for unmarried opposite-sex couples.<br>• The typical ZIP code characteristics of male same-sex couples show a greater percentage of gay male couples than in typical married opposite-sex couple neighborhoods.<br>• The typical ZIP code characteristics of male same-sex couples show a lower percentage of married couples than in typical unmarried opposite-sex couple neighborhoods.<br>• The typical ZIP code characteristics of male same-sex couples show a lower percentage of unmarried couples than in typical unmarried opposite-sex couple neighborhoods.<br>• The typical ZIP code characteristics of male same-sex couples show higher median individual income than for unmarried opposite-sex couples. (p. 36)<br>• The typical ZIP code characteristics of male same-sex couples show higher rates of college graduates than for opposite-sex couples. (p. 36)<br>• The typical ZIP code characteristics of male same-sex couples show higher rates of foreign born residents than for opposite-sex couples. (p. 36)<br>• The typical ZIP code characteristics of male same-sex couples show higher rates of non-English speaking residents than for opposite-sex couples. (p. 36)<br>• The typical ZIP code characteristics of male same-sex couples show higher share of homes built before 1939 than for married opposite-sex couples.<br>• The typical ZIP code characteristics of male same-sex couples show higher share of homes built before 1939 than for unmarried opposite-sex couples.<br>• The typical ZIP code characteristics of male same-sex couples show no difference in median individual income than from married opposite-sex couples. (p. 36)

RoB *	Author 1 Year	Study Design, Year, N of individuals/couples	Sampling Frame: Sampling Strategy; definition of sexual orientation	Study area [Country: Location, Area Unit(s) of Analysis, N of area units]	Type of Quantitative Analysis Utilized	**Outcome(s)** (Bold indicates inclusion in Harvest Plot)
3	(Gates, 2013)	Cross-sectional, 2009, NR	Adult households: American Community Survey, Survey, same-sex couples	U.S., 1: Urban/rural; 2: Metropolitan Area, NR	Descriptive	• 16% of the same-sex couples lived in rural areas (men 14%; women 19%) compared to 23% of the population<br>• 44% of the same-sex couples lived in metropolitan areas, outside principal cities (men 40%; women 48%) compared to 51% of the population<br>• 46% of the same-sex couples lived in metropolitan areas, principal cities (men 51%; women 41%) compared to 33% of the population<br>• 10% of the same-sex couples lived in non-metropolitan areas (men 8%; women 11%) compared to 16% of the population<br>• 84% of the same-sex couples lived in urban areas (men 86 %; women 81 %) compared to 77% of the population
2	(Giraud, 2011)	Cross-sectional, 2007, 727	All subscribers to the LGBT magazine *Têtu*, total, Subscriber of *Têtu*	France, Paris, residential areas (arrondissements), 20	Descriptive	• 23,0 % of the subscribers lived in "Desirable areas" λ=1,2-2<br>• Segregation index of gay male periodical subscriber list to all men in Paris (area units: arrondissement): 0.23 (in 1997)<br>• Segregation index of gay male periodical subscriber list to all men in Paris (area units: arrondissement): 0.21 (in 2007)<br>• 11,3 % of the subscribers lived in "Highly desirable areas" λ>2<br>• 20.6 % of the subscribers lived in "Undesirable areas" λ=0.5-0.9<br>• 37.6 % of the subscribers lived in "Neutral areas" λ=0.9-1.2<br>• 7.5 % lived in "Highly desirable areas" λ=<0.5<br>• Segregation index of gay male periodical subscriber list to all men in Paris (area units: arrondissement): 0.20 (in 2002)
2	(Guo et al., 2016)	Cross-sectional, 2009, 22288 (1454 gay, lesbian, bisexual, or "unsure")	Nationally representative sample of unmarried youth age 15-24, identity (Q: "What is your sexual orientation?")	China	Logistic regression	• In logistic regression adjusted for age, region, mother's education, family income, and only child, non-heterosexual status was positively associated with rural residence, OR 1.23 (95% CI: 1.09-1.40); however, this was largely driven by answers of "unsure" to the question.<br>• In unadjusted logistic regression, non-heterosexual status was positively associated with rural residence, OR 1.36 (95% CI: 1.22-1.51); however, this was driven largely by answers of "unsure" to the question.<br>• The percentage of homosexual young people living in urban areas was higher (0.86%) than in rural areas (0.70%).<br>• The percentage of bisexual young people living in urban areas was lower (1.27%) than in rural areas (1.74%).

RoB*	Author 1 Year	Study Design, Year, N of individuals/couples	Sampling Frame: Sampling Strategy; definition of sexual orientation	Study area [Country: Location, Area Unit(s) of Analysis, N of area units]	Type of Quantitative Analysis Utilized	**Outcome(s)** (Bold indicates inclusion in Harvest Plot)
4	(Hayslett & Kane, 2011)	Cross-Sectional, 2000, 1,585 gay couples and 1,348 lesbian couples	Columbus, OH, household: census; Census derived same-sex households (DV is calculated as the same-sex couple percentage in a tract to the same-sex couple percentage in the city)	Columbus, OH, census tracts, 226	Spatial regression (error models for same-sex female couples, lag model for same-sex male couples)	• In an adjusted model (for other neighborhood amenities and housing characteristic variables), the percentage of renters is associated with same-sex male concentration, b=0.01, p<0.05<br>• In an adjusted model (for other neighborhood amenities and housing characteristic variables), the cultural amenities index is marginally associated with same-sex male concentration, b=0.25, p<0.10<br>• In an adjusted model (for other neighborhood amenities and housing characteristic variables), the cultural amenities index was marginally associated with the concentration of same-sex male couples, b=0.33, p<0.10<br>• In an adjusted model (for other neighborhood composition variables), tract % of households renting was associated with the concentration of same-sex male couples, b=0.01, p<0.01<br>• In an adjusted model (for other neighborhood amenities and housing characteristic variables), the concentration of gays and lesbians is associated with same-sex male concentration, b=0.54, p<0.001<br>• In an adjusted model (for other neighborhood composition variables), tract % foreign born was not associated with the concentration of male same-sex couples, b=6.23, p>0.10<br>• In an adjusted model (for other neighborhood amenities and housing characteristic variables), the average housing value per sq. ft. was associated with the concentration of same-sex male couples, b=0.01, p<0.01<br>• In an adjusted model (for other neighborhood amenities and housing characteristic variables), the cultural amenities index was not associated with the concentration of same-sex female couples, b=0.04, p>0.10<br>• In an adjusted model (for other neighborhood amenities and housing characteristic variables), the median year housing was built is not associated with same-sex male concentration, b=-0.00, p>0.10<br>• In an adjusted model (for other neighborhood amenities and housing characteristic variables), the tract percentage of children is associated with same-sex male concentration, b=-0.03, p<0.001<br>• In an adjusted model (for other neighborhood composition variables), tract gay/lesbian concentration was associated with the concentration of male same-sex couples, b=0.60, p<0.001<br>• In an adjusted model (for neighborhood composition, amenities, and housing characteristics), the % of residents with a college degree is not associated with the same-sex male concentration, b=0.69, p>0.10<br>• In an adjusted model (for other neighborhood amenities and housing characteristic variables), the % foreign born is marginally associated

RoB*	Author 1 Year	Study Design, Year, N of individuals/couples	Sampling Frame: Sampling Strategy; definition of sexual orientation	Study area [Country: Location, Area Unit(s) of Analysis, N of area units]	Type of Quantitative Analysis Utilized	**Outcome(s)** (Bold indicates inclusion in Harvest Plot)
						with the same-sex female concentration, b=16.64, p<0.10<br>• In an adjusted model (for other neighborhood amenities and housing characteristic variables), the count of malls and shopping centers is negatively associated with the concentration of same-sex male couples, b=-0.09, p<0.05<br>• In an adjusted model (for other neighborhood amenities and housing characteristic variables), the presence of a gay bar within 1 mile of the tract centroid is not associated with the concentration of same-sex female couples, b=0.10, p>0.10<br>• In an adjusted model (for other neighborhood amenities and housing characteristic variables), the presence of a gay bar within 1 mile of the tract centroid is not associated with the concentration of same-sex male couples, b=0.05, p>0.10<br>• In an adjusted model (for other neighborhood composition variables), tract gay/lesbian concentration was associated with the concentration of female same-sex couples, b=0.28, p<0.001<br>• In an adjusted model (for other neighborhood amenities and housing characteristic variables), the average value per sq. ft. is marginally associated with same-sex female concentration, b=0.01, p<0.10<br>• In an adjusted model (for other neighborhood amenities and housing characteristic variables), the concentration of gays and lesbians is associated with same-sex female concentration, b=0.24, p<0.001<br>• In an adjusted model (for other neighborhood amenities and housing characteristic variables), the count of malls and shopping centers is associated with same-sex male concentration, b=-0.08, p<0.10<br>• In an adjusted model (for other neighborhood amenities and housing characteristic variables), the median year housing was built was associated with same-sex female concentration, b=-0.01, p<0.001<br>• In an adjusted model (for other neighborhood amenities and housing characteristic variables), the percent children is associated with the same-sex female concentration, b=0.01, p<0.05<br>• In an adjusted model (for other neighborhood composition variables), tract % black residents was not associated with the concentration of female same-sex couples, b=0.00, p>0.10<br>• In an adjusted model (for other neighborhood amenities and housing characteristic variables), the average value per sq. ft. is not associated with same-sex male concentration, b=-0.00, p>0.10<br>• In an adjusted model (for other neighborhood amenities and housing characteristic variables), the count of malls and shopping centers is not associated with the concentration of same-sex female couples, b=-0.01, p>0.10<br>• In an adjusted model (for other neighborhood amenities and housing

RoB *	Author 1 Year	Study Design, Year, N of individuals/couples	Sampling Frame: Sampling Strategy; definition of sexual orientation	Study area [Country: Location, Area Unit(s) of Analysis, N of area units]	Type of Quantitative Analysis Utilized	**Outcome(s)** (Bold indicates inclusion in Harvest Plot)
						characteristic variables), the median year of house construction was associated with the concentration of same-sex female households, b=-0.02, p<0.001<br>• In an adjusted model (for other neighborhood amenities and housing characteristic variables), the percent of housing units that are single family was associated with the concentration of same-sex male couples, b=-0.00, p<0.05<br>• In an adjusted model (for other neighborhood amenities and housing characteristic variables), the percent of housing units that are single family was not associated with the concentration of same-sex female couples, b=-0.00, p>0.10<br>• In an adjusted model (for other neighborhood amenities and housing characteristic variables), the percentage of housing for multifamily use is not associated with same-sex male concentration, b=0.01, p>0.10<br>• In an adjusted model (for other neighborhood amenities and housing characteristic variables), the percentage of housing units that are multifamily was associated with the same-sex female concentration, b=0.02, p<0.01<br>• In an adjusted model (for other neighborhood composition variables), tract % age 18-24 was not associated with the same-sex female concentration, b=-0.01, p>0.10<br>• In an adjusted model (for other neighborhood composition variables), tract % children was associated with the same-sex female concentration, b=0.01, p<0.05<br>• In an adjusted model (for other neighborhood composition variables), tract % foreign born was associated with the concentration of female same-sex couples, b=19.84, p<0.05<br>• In an adjusted model (for other neighborhood amenities and housing characteristic variables), the percentage of housing units that are multifamily was associated with the same-sex male concentration, b=0.02, p<0.05<br>• In an adjusted model (for other neighborhood composition variables), tract % age 18-24 was associated with the same-sex male concentration, b=-0.02, p<0.01<br>• In an adjusted model (for other neighborhood composition variables), tract % children was associated with the same-sex male concentration, b=-0.03, p<0.001<br>• In an adjusted model (for other neighborhood composition variables), tract % college degree was not associated with the concentration of male same-sex couples, b=0.79, p<0.10<br>• In an adjusted model (for other neighborhood amenities and housing

RoB*	Author 1 Year	Study Design, Year, N of individuals/couples	Sampling Frame: Sampling Strategy; definition of sexual orientation	Study area [Country: Location, Area Unit(s) of Analysis, N of area units]	Type of Quantitative Analysis Utilized	**Outcome(s)** (Bold indicates inclusion in Harvest Plot)
						characteristic variables), the average housing value per sq. ft. was associated with the concentration of same-sex female couples, b=0.01, p<0.05<br>• In an adjusted model (for other neighborhood amenities and housing characteristic variables), the median year of house construction was associated with the concentration of same-sex male households, b=-0.02, p<0.05<br>• In an adjusted model (for other neighborhood amenities and housing characteristic variables), the number of schools is not associated with the same-sex female concentration, b=0.03, p>0.10<br>• In an adjusted model (for other neighborhood amenities and housing characteristic variables), the number of schools is not associated with the same-sex male concentration, b=0.06, p>0.10<br>• In an adjusted model (for other neighborhood amenities and housing characteristic variables), the percentage of housing for multifamily use is associated with same-sex female concentration, b=0.01, p<0.05<br>• In an adjusted model (for other neighborhood amenities and housing characteristic variables), the tract % age 18-24 is associated with the male same-sex couple concentration, b=-0.03, p<0.01<br>• In an adjusted model (for other neighborhood composition variables), tract % black residents was not associated with the concentration of male same-sex couples, b=0.00, p>0.10<br>• In an adjusted model (for other neighborhood composition variables), tract % college degree was not associated with the concentration of female same-sex couples, b=-0.08, p>0.10<br>• In an adjusted model (for other neighborhood composition variables), tract % of households renting was not associated with the concentration of same-sex female couples, b=-0.00, p>0.10
1	(Hughes & Saxton, 2006)	Cross-Sectional 1996 New Zealand Census (2,883 males in a same-sex cohabiting couple) vs. 1,852 Male Call non-representative survey in NZ	Census and Male Call Phone Survey: Census and self-selected convenience sample; same-sex couples and behavior	New Zealand, NR, NR	Descriptive	• In Auckland, 13.6% of census males, 9.7% of opposite-sex couples, but 41.9% of same-sex male couples and 44.6% of male call gay men lived in the Inner City District.<br>• There were fewer census and Male call same-sex couples and gay men in minor urban areas, 4.2% and 3.8%, than opposite sex census males (8.5%) or census opposite-sex couples (8.6%).<br>• In Auckland, 86.4% of census males, 90.3% of opposite-sex couples, but 58.1% of same-sex male couples and 55.4% of male call gay men lived in the Inner City District.<br>• There were fewer census male same-sex couples and Male call gay men in “other main urban areas,” 9.4 and 12.2 respectively, than census males (16.0) or census opposite sex couples (15.9).<br>• In Auckland, 35.1% of census males, 31.7% of opposite-sex

RoB*	Author 1 Year	Study Design, Year, N of individuals/couples	Sampling Frame: Sampling Strategy; definition of sexual orientation	Study area [Country: Location, Area Unit(s) of Analysis, N of area units]	Type of Quantitative Analysis Utilized	**Outcome(s)** (Bold indicates inclusion in Harvest Plot)
						couples, but 64.6% of same-sex male couples and 66.3% of male call gay men lived in the Central Zone.<br>• There were fewer census and Male Call same-sex couples and gay men in rural areas 9.4 and 2.4, respectively, than in the Census (15.0% single men, 16.1% opposite sex couples).<br>• There were fewer census male same-sex couples and Male call gay men in secondary urban areas, 2.4 and 3.3 respectively, than census males (7.3) or census opposite sex couples (7.7).
4	(Klein & Smart, 2016)	Cross-sectional, 2007-2011 ACS PUMS and National Household Travel Survey, ACS: 4032537 (22293 same-sex males and 23292 same-sex females) and NHTS: 151437 (527 same-sex males and 560 same-sex females)	USA: Census (ACS) and probability based (NHTS); same-sex cohabitation	USA, PUMA (ACS) and tract (NHTS)	Descriptive	• **In unadjusted relationship between same-sex male households and opposite-sex households in NHTS data, there is a significantly higher employee density for same-sex male households at the PUMA area unit, M=12156 vs. 1605, p<0.01.**<br>• **In unadjusted relationship between same-sex female households and opposite-sex households in ACS data, there is a significantly higher employee density for same-sex female households at the PUMA area unit, M=3275 vs. 1679, p<0.01.**<br>• **In unadjusted relationship between same-sex female households and opposite-sex households in ACS data, there is a significantly higher population density for same-sex female households at the PUMA area unit, M=3735 vs. 2540, p<0.01.**<br>• **In unadjusted relationship between same-sex male households and opposite-sex households in ACS data, there is a significantly higher employee density for same-sex male households at the PUMA area unit, M=9076 vs. 1731, p<0.01.**<br>• **In unadjusted relationships in NHTS data, the distribution of metro region categories was not more urban for female same-sex couples than opposite-sex couples, p>0.05**<br>• **In unadjusted relationship between same-sex male households and opposite-sex households in NHTS data, there is a significantly higher average tract percentage of same-sex coupled households at the PUMA area unit for same-sex male couples, M=1.14 vs. 0.60, p<0.01.**<br>• In unadjusted relationship between **same-sex male households** and opposite-sex households in NHTS data, there is a significantly higher mean percentage of buildings built before 1950 density for same-sex male households at the PUMA area unit, M=28.8 vs. 17.5, p<0.05.<br>• In unadjusted relationship between same-sex female households and opposite-sex households in ACS data, there is a significantly higher mean percentage of buildings built before 1950 density for same-sex female households at the PUMA area unit, M=21.3 vs. 18.4, p<0.01.

RoB*	Author 1 Year	Study Design, Year, N of individuals/couples	Sampling Frame: Sampling Strategy; definition of sexual orientation	Study area [Country: Location, Area Unit(s) of Analysis, N of area units]	Type of Quantitative Analysis Utilized	**Outcome(s)** (Bold indicates inclusion in Harvest Plot)
						• **In unadjusted relationship between same-sex female households and opposite-sex households in NHTS data, there is no association population density at the PUMA area unit, M=3233 vs. 3955, p>0.05.**<br>• In unadjusted relationship between **same-sex male households** and opposite-sex households in ACS data, there is a significantly higher mean percentage of buildings built before 1950 density for same-sex male households at the PUMA area unit, M=24.8 vs. 18.0, p<0.01.<br>• **In unadjusted relationships in ACS data, the distribution of metro region categories was significantly more urban for male same-sex couples than opposite-sex couples, p<0.01**<br>• **In unadjusted relationship between same-sex male households and opposite-sex households in ACS data, there is a significantly higher population density for same-sex male households at the PUMA area unit, M=6482 vs. 2656, p<0.01.**<br>• **In unadjusted relationship between same-sex male households and opposite-sex households in NHTS data, there is a significantly higher population density at the PUMA area unit, M=9301 vs. 3947, p<0.01.**<br>• **In unadjusted relationships in ACS data, the distribution of metro region categories was significantly more urban for female same-sex couples than opposite-sex couples, p<0.01**<br>• **In unadjusted relationship between same-sex female households and opposite-sex households in NHTS data, there is a not an association with the percentage of same-sex coupled households in the tracts at the PUMA area unit, M=0.85 vs. 0.60, p>0.05.**<br>• **In unadjusted relationship between same-sex female households and opposite-sex households in NHTS data, there is no association with employee density for same-sex male households at the PUMA area unit, M=1778 vs. 1607, p>0.05.**<br>• **In unadjusted relationships in NHTS data, the distribution of metro region categories was significantly more urban for male same-sex couples than opposite-sex couples, p<0.01**<br>• There are differences in travel (work commute) patterns by the concentration of same-sex couples in a census tract.
3	(Laumann, Gagnon, Michael, & Michaels, 1994)	Cross-sectional (with retrospective report), 1988-1991 and 1993, ~4800	USA: probability-based (General Social Survey and NHSLS); Reporting any same gender partners	USA, NR, NR	Descriptive	• Among men and to a lesser extent women reporting same gender partners in the last year, past five years, and since age 18, there is a general trend of living in more urban areas. (see Laumann's Table 8.1 for details)<br>• Among men and to a lesser extent women reporting same gender

RoB*	Author 1 Year	Study Design, Year, N of individuals/couples	Sampling Frame: Sampling Strategy; definition of sexual orientation	Study area [Country: Location, Area Unit(s) of Analysis, N of area units]	Type of Quantitative Analysis Utilized	**Outcome(s)** (Bold indicates inclusion in Harvest Plot)
						partners in the last year, past five years, and since age 18, there is a general trend towards living in more urban areas at age 14/16 (retrospectively reported) (see Laumann's Table 8.1 for details)<br>• Among men and to a lesser extent women reporting any domain of sexual orientation in the last year, past five years, and since age 18, there is a general trend of living in more urban areas. (see Laumann's Table 8.2 for details)<br>• Among men and to a lesser extent women reporting any domain of sexual orientation in the last year, past five years, and since age 18, there is a general trend towards living in more urban areas at age 14/16 (see Laumann's Table 8.2 for details
3	(Lee, Goldstein, Pan, & Ribisl, 2015)	Cross-sectional, 2010 (Census Data), 2012 (retailer marketing data), NR	Census: Census (same-sex couples) and random (tobacco retailers); derived same-sex household	U.S. (97 unique counties), census tracts, 1696	Multilevel regression models	• **In an unadjusted model, the same-sex female rate was not associated with advertised price of Marlboro Red cigarettes in retailers, b=<$0.01, p>0.05**<br>• In a model adjusted for neighborhood and store characteristics, the count of exterior tobacco marketing materials at tobacco retailers was not associated with the census tract same-sex female couple rate, OR 1.00 (0.98-1.01)<br>• In a model adjusted for neighborhood and store characteristics, the presence of a price promotions at tobacco retailers was not associated with the census tract same-sex female couple rate, OR 1.00 (0.99-1.00)<br>• In a model adjusted for neighborhood characteristics, the presence flavored cigars at tobacco retailers was not associated with the census tract same-sex female couple rate, OR 0.99 (0.99-1.00)<br>• In a model adjusted for neighborhood and store characteristics, the count of exterior tobacco marketing materials at tobacco retailers was not associated with the census tract same-sex female couple rate, OR 1.00 (0.99-1.00)<br>• In a model adjusted for neighborhood characteristics, the presence of a *Newport* price promotions at tobacco retailers was not associated with the census tract same-sex female couple rate, OR 0.98 (0.96-1.01)<br>• In a model adjusted for neighborhood characteristics, the presence of a *Newport* price promotions at tobacco retailers was not associated with the census tract same-sex female couple rate, OR 0.99 (0.99-1.00)<br>• **The presence of a price promotions at tobacco retailers was not associated with the census tract same-sex male couple rate, OR 1.00 (0.99-1.00)**<br>• In a model adjusted for neighborhood and store characteristics, the presence of a e-cigarettes at tobacco retailers was not associated

RoB*	Author 1 Year	Study Design, Year, N of individuals/couples	Sampling Frame: Sampling Strategy; definition of sexual orientation	Study area [Country: Location, Area Unit(s) of Analysis, N of area units]	Type of Quantitative Analysis Utilized	**Outcome(s)** (Bold indicates inclusion in Harvest Plot)
						with the census tract same-sex female couple rate, OR 1.00 (0.98-1.02)<br>• In a model adjusted for neighborhood and store characteristics, the presence of a price promotions at tobacco retailers was not associated with the census tract same-sex female couple rate, OR 1.00 (0.97-1.02)<br>• In a model adjusted for neighborhood characteristics, the presence of a e-cigarettes at tobacco retailers was not associated with the census tract same-sex female couple rate, OR 0.99 (0.97-1.01)<br>• In a model adjusted for neighborhood characteristics, the presence of a price promotions at tobacco retailers was not associated with the census tract same-sex female couple rate, OR 0.99 (0.96-1.01)<br>• In an model adjusted for neighborhood and store characteristics, the same-sex male rate was associated with advertised price of Newport Green cigarettes in retailers, b=<$0.01, p<0.05<br>• In an model adjusted for neighborhood characteristics, the same-sex female rate was associated with advertised price of Newport Green cigarettes in retailers, b=$0.01, p<0.05<br>• In an model adjusted for neighborhood characteristics, the same-sex female rate was not associated with advertised price of Marlboro Red cigarettes in retailers, b=<$0.01, p>0.05<br>• In an model adjusted for neighborhood characteristics, the same-sex male rate was not associated with advertised price of Marlboro Red cigarettes in retailers, b=<$0.01, p>0.05<br>• **The count of tobacco marketing materials at tobacco retailers was negatively associated with the census tract same-sex female couple rate, OR 0.99 (0.98-1.00), p<0.05**<br>• In a model adjusted for neighborhood and store characteristics, the count of tobacco marketing materials at tobacco retailers was negatively associated with the census tract same-sex female couple rate, OR 1.00 (1.00-1.00), p<0.05<br>• In a model adjusted for neighborhood and store characteristics, the presence flavored cigars at tobacco retailers was negatively associated with the census tract same-sex female couple rate, OR 0.99 (0.99-1.00), p<0.05<br>• In a model adjusted for neighborhood and store characteristics, the presence of a *Newport* price promotions at tobacco retailers was not associated with the census tract same-sex female couple rate, OR 0.99 (0.96-1.01)<br>• In a model adjusted for neighborhood characteristics, the count of tobacco marketing materials at tobacco retailers was negatively associated with the census tract same-sex female couple rate, OR

RoB*	Author 1 Year	Study Design, Year, N of individuals/couples	Sampling Frame: Sampling Strategy; definition of sexual orientation	Study area [Country: Location, Area Unit(s) of Analysis, N of area units]	Type of Quantitative Analysis Utilized	**Outcome(s)** (Bold indicates inclusion in Harvest Plot)
						0.99 (0.98-1.00), p<0.05<br>• In a model adjusted for neighborhood characteristics, the presence of e-cigarettes at tobacco retailers was not associated with the census tract same-sex female couple rate, OR 1.00 (1.00-1.01)<br>• In a model adjusted for neighborhood characteristics, the presence of a price promotions at tobacco retailers was not associated with the census tract same-sex female couple rate, OR 1.00 (0.99-1.00)<br>• In an model adjusted for neighborhood characteristics, the same-sex male rate was associated with advertised price of Newport Green cigarettes in retailers, b=<$0.01, p<0.05<br>• **In an unadjusted model, the same-sex female rate was not associated with advertised price of Newport Green cigarettes in retailers, b=<$0.01, p>0.05**<br>• **In an unadjusted model, the same-sex male rate was associated with advertised price of Newport Green cigarettes in retailers, b=<$0.01, p<0.05**<br>• **In an unadjusted model, the same-sex male rate was not associated with advertised price of Marlboro Red cigarettes in retailers, b=<$0.01, p>0.05**<br>• **The count of exterior tobacco marketing materials at tobacco retailers was not associated with the census tract same-sex female couple rate, OR 1.00 (0.99-1.02)**<br>• **The presence of a e-cigarettes at tobacco retailers was not associated with the census tract same-sex female couple rate, OR 0.99 (0.97-1.01)**<br>• **The presence of a *Newport* price promotions at tobacco retailers was not associated with the census tract same-sex male couple rate, OR 1.00 (0.99-1.01)**<br>• In a model adjusted for neighborhood and store characteristics, the count of tobacco marketing materials at tobacco retailers was negatively associated with the census tract same-sex female couple rate, OR 0.99 (0.98-1.00), p<0.05<br>• In a model adjusted for neighborhood and store characteristics, the presence of e-cigarettes at tobacco retailers was not associated with the census tract same-sex female couple rate, OR 1.00 (0.99-1.00)<br>• In a model adjusted for neighborhood and store characteristics, the same-sex male rate was associated with advertised price of Marlboro Red cigarettes in retailers, b=<$0.01, p<0.05<br>• In a model adjusted for neighborhood characteristics, the count of exterior tobacco marketing materials at tobacco retailers was negatively associated with the census tract same-sex female couple

RoB*	Author 1 Year	Study Design, Year, N of individuals/couples	Sampling Frame: Sampling Strategy; definition of sexual orientation	Study area [Country: Location, Area Unit(s) of Analysis, N of area units]	Type of Quantitative Analysis Utilized	**Outcome(s)** (Bold indicates inclusion in Harvest Plot)
						rate, OR 0.99 (0.99-1.00), p<0.05<br>• In a model adjusted for neighborhood characteristics, the presence flavored cigars at tobacco retailers was not associated with the census tract same-sex female couple rate, OR 0.99 (0.97-1.01)<br>• **The presence of a *Newport* price promotions at tobacco retailers was not associated with the census tract same-sex female couple rate, OR 1.01 (0.99-1.03)**<br>• In a model adjusted for neighborhood and store characteristics, the presence flavored cigars at tobacco retailers was not associated with the census tract same-sex female couple rate, OR 0.99 (0.97-1.02)<br>• In a model adjusted for neighborhood and store characteristics, the presence of a *Newport* price promotions at tobacco retailers was not associated with the census tract same-sex female couple rate, OR 0.99 (0.99-1.00)<br>• In a model adjusted for neighborhood and store characteristics, the same-sex female rate was associated with advertised price of Newport Green cigarettes in retailers, b=$0.01, p<0.05<br>• In a model adjusted for neighborhood and store characteristics, the same-sex female rate was not associated with advertised price of Marlboro Red cigarettes in retailers, b=<$0.01, p>0.05<br>• **The presence flavored cigars at tobacco retailers was not associated with the census tract same-sex female couple rate, OR 1.01 (0.98-1.03)**<br>• **The presence of a price promotions at tobacco retailers was not associated with the census tract same-sex female couple rate, OR 1.00 (0.98-1.02)**<br>• **The count of tobacco marketing materials at tobacco retailers was negatively associated with the census tract same-sex male couple rate, OR 1.00 (0.99-1.00), p<0.05**<br>• **The presence flavored cigars at tobacco retailers was not associated with the census tract same-sex male couple rate, OR 1.00 (0.99-1.00)**<br>• **The presence of a e-cigarettes at tobacco retailers was not associated with the census tract same-sex male couple rate, OR 1.00 (1.00-1.01)**<br>• In a model adjusted for neighborhood characteristics, the count of exterior tobacco marketing materials at tobacco retailers was not associated with the census tract same-sex female couple rate, OR 0.99 (0.97-1.01)<br>• In a model adjusted for neighborhood characteristics, the count of tobacco marketing materials at tobacco retailers was negatively

RoB *	Author 1 Year	Study Design, Year, N of individuals/couples	Sampling Frame: Sampling Strategy; definition of sexual orientation	Study area [Country: Location, Area Unit(s) of Analysis, N of area units]	Type of Quantitative Analysis Utilized	**Outcome(s)** (Bold indicates inclusion in Harvest Plot)
						associated with the census tract same-sex female couple rate, OR 1.00 (0.99-1.00), p<0.05<br>• **The count of exterior tobacco marketing materials at tobacco retailers was not associated with the census tract same-sex male couple rate, OR 0.99 (0.99-1.00)**
3	(Lee, Pan, Henriksen, Goldstein, & Ribisl, 2016)	Cross-sectional Study, 2012 (Census 2010; 2008-2012), N/A	97 U.S. Counties: Census of tracts with >250 households; same-sex couples per 1000 coupled households	USA: 97 U.S. Counties, census tracts, 17,667	Spatial regression	• **In unadjusted spatial regression, the rate of male same-sex couples was associated with the number of tobacco retailers per 1,000 population in census tracts, b=0.01, p<0.001.**<br>• **In unadjusted spatial regression, the rate of female same-sex couples was associated with the number of tobacco retailers per 1,000 population in census tracts, b=0.01, p<0.001.**<br>• For female but not male same-sex couples this relationship was explained by other neighborhood demographics (% Black, % Hispanic, income).
2	(Marcus, Schmidt, Hamouda, & Bochow, 2009)	Cross-sectional, 2006/2007/2008; online surveys 5358 and 8170, online data 290 000	MSM targeting; sexual identity; participation in online surveys, participation in gay dating web site	Germany: Regions	Descriptive	• "These MSM concentrations in the largest German cities are counterbalanced by a lower concentration (concentration factor < 1.0) of MSM in many rural regions, especially in southern, eastern and central parts of the country. If the regional distribution of MSM is stratified by age, the concentration factors in the larger cities disappear for the age group of MSM younger than 26 years (data not shown)" (p. 7)
4	(Matthews & Besemer, 2015)	Cross-sectional, pooled 2008-2011 Scottish Health Survey, n=24,837	Scottish Households: Stratified Random: "Which of the following best describes your sexual orientation? (If forming any of the following relationships: girlfriend/boyfriend/wife/husband/ partner – with which sex(es) would that be?). Tick ONE box". The options are: Bisexual (both sexes); Gay or Lesbian (same sex); Heterosexual (opposite sex); Other; and until 2009, a Prefer not to answer category. (p. 100)	Great Britain: Scotland, datazones, NR	Descriptive/Logistic regressions	• 22% of LGB or Other respondents lived in the second poorest SIMD quintile.<br>• 24% of LGB or Other respondents lived in the poorest SIMD quintile.<br>• In an unadjusted model, the odds of living in the poorest SIMD quintile were 42% higher for LGB or Other identifying respondents compared to heterosexual identifying respondents.<br>• In an adjusted model controlling for relationship status, presence of children, bad health, limiting illness, being a homeowner, and having low income, LGB or Other identity did not predict the likelihood of living in a poorer area.<br>• In an unadjusted model, the odds of living in the fourth poorest SIMD quintile were 24% lower for LGB or Other identifying respondents compared to heterosexual identifying respondents, p<0.01<br>• 17% of LGB or other respondents lived in the fourth poorest SIMD quintile.<br>• **In an unadjusted model, the odds of living in the least poor SIMD quintile were 24% lower for LGB or Other identifying respondents compared to heterosexual identifying**

RoB*	Author 1 Year	Study Design, Year, N of individuals/couples	Sampling Frame: Sampling Strategy; definition of sexual orientation	Study area [Country: Location, Area Unit(s) of Analysis, N of area units]	Type of Quantitative Analysis Utilized	**Outcome(s)** (Bold indicates inclusion in Harvest Plot)
						**respondents.**<br>• In an unadjusted model, the odds of living in the second poorest SIMD quintile were 9% higher for LGB or Other identifying respondents compared to heterosexual identifying respondents, NS<br>• In an unadjusted model, the odds of living in the third poorest SIMD quintile were 3% higher for LGB or Other identifying respondents compared to heterosexual identifying respondents, NS<br>• 16% of LGB or other respondents lived in the fifth poorest SIMD quintile<br>• 20% of LGBT or Other respondents lived in the third poorest SIMD quintile
3	(Minnis et al., 2016)	Cross-sectional, 2013-2014, 21322 (751 LGB)	Adults, Probability-based; identity	20 U.S. sites of Community Transformation Grants (Adult Targeted Surveillance Survey, ATSS), NR	Descriptive	• Participants reported some differences by gender and sexual orientation in the availability of fruit and vegetables in their communities (Straight men 75.9 %, Gay men 71.2 %, Bisexual men 69.1 %, Straight women 72.3 %, Lesbians 68.8 %, Bisexual women 75.3 %, p=.02), the walking and cycling environment (mean: Straight men 2.6, Gay men 3.1, Bisexual men 2.7, Straight women 2.5, Lesbians 3.1, Bisexual women 2.8, p<.00), and rural status at the county level (Straight men 18.5 %, Gay men 5.0 %, Bisexual men 9.8 %, Straight women 18.8 %, Lesbians 9.7 %, Bisexual women 16.0 %, p<.001), % of census tract population below poverty (Straight men 18.2 %, Gay men 22.8 %, Bisexual men 20.0 %, Straight women 19.3 %, Lesbians 21.8 %, Bisexual women 24.8 %, p<.001), population with less than high school education (Straight men 17.0 %, Gay men 17.1 %, Bisexual men 17.3 %, Straight women 17.8 %, Lesbians 20.5 %, Bisexual women 20.5 %, p=.08), and no clear differences in census tract % employed (Straight men 89.7 %, Gay men 88.3 %, Bisexual men 89.9 %, Straight women 89.2 %, Lesbians 89.2 %, Bisexual women 88.0 %, p=.01. *No statistical test compared LGB vs. heterosexual respondents.*
4	(Rosenfeld, 2007)	Cross-sectional, 1990 and 2000, 1990 (NR) and 2000 (2,706,642)	USA: Census (5% IPUMS microdata); cohabiting same-sex couples	USA, NR, NR	Descriptive	• Among couples living in rural areas, in unadjusted logistic regression, same-sex couples have 1.50 greater likelihood of living in a different state than their birth than heterosexual, married, same-race couples, p<0.001, in 1990 data. (p. 199)<br>• Among couples living in suburban areas, in unadjusted logistic regression, same-sex couples have 1.75 greater likelihood of living in a different state than their birth than heterosexual, married, same-race couples, p<0.001, in 1990 data. (p. 199)<br>• Among couples living in urban areas, in unadjusted logistic regression, same-sex interracial couples have 2.50 greater likelihood of living in a different state than their birth than heterosexual, married, same-race couples, p<0.001, in 2000 data.

RoB*	Author 1 Year	Study Design, Year, N of individuals/couples	Sampling Frame: Sampling Strategy; definition of sexual orientation	Study area [Country: Location, Area Unit(s) of Analysis, N of area units]	Type of Quantitative Analysis Utilized	**Outcome(s)** (Bold indicates inclusion in Harvest Plot)
						(p. 199)<br>• In adjusted logistic regression controlling for education, mobility, age, and living in a city, percent of gay men in the metro area predicted odds of same-sex cohabitation, OR 1.81, p<0.001, in 2000 data (p. 201)<br>• Among couples living in rural areas, in unadjusted logistic regression, same-sex interracial couples have 2.94 greater likelihood of living in a different state than their birth than heterosexual, married, same-race couples, p<0.001, in 1990 data. (p. 199)<br>• Among couples living in rural areas, in unadjusted logistic regression, same-sex interracial couples have 3.13 greater likelihood of living in a different state than their birth than heterosexual, married, same-race couples, p<0.001, in 2000 data. (p. 199)<br>• Among couples living in rural areas, in unadjusted logistic regression, same-sex couples have 1.17 greater likelihood of living in a different state than their birth than heterosexual, married, same-race couples, p<0.001, in 2000 data. (p. 199)<br>• Among couples living in suburban areas, in unadjusted logistic regression, same-sex interracial couples have 1.91 greater likelihood of living in a different state than their birth than heterosexual, married, same-race couples, p<0.001, in 2000 data. (p. 199)<br>• Among couples living in urban areas, in unadjusted logistic regression, same-sex couples have 1.76 greater likelihood of living in a different state than their birth than heterosexual, married, same-race couples, p<0.001, in 2000 data. (p. 199)<br>• Among couples living in urban areas, in unadjusted logistic regression, same-sex couples have 2.76 greater likelihood of living in a different state than their birth than heterosexual, married, same-race couples, p<0.001, in 1990 data. (p. 199)<br>• Among couples living in urban areas, in unadjusted logistic regression, same-sex interracial couples have 2.73 greater likelihood of living in a different state than their birth than heterosexual, married, same-race couples, p<0.001, in 1990 data. (p. 199)<br>• **In unadjusted models predicting odds of urban residence, young white same-sex couples had 2.36 times the likelihood of living in an urban area than white heterosexual couples, p<0.001, in 2000 data (p. 198)**<br>• Among couples living in suburban areas, in unadjusted logistic

RoB *	Author 1 Year	Study Design, Year, N of individuals/couples	Sampling Frame: Sampling Strategy; definition of sexual orientation	Study area [Country: Location, Area Unit(s) of Analysis, N of area units]	Type of Quantitative Analysis Utilized	**Outcome(s)** (Bold indicates inclusion in Harvest Plot)
						regression, same-sex couples have 1.31 greater likelihood of living in a different state than their birth than heterosexual, married, same-race couples, p<0.001, in 2000 data. (p. 199)<br>• In adjusted logistic regression controlling for education, mobility, age, and percent of gay men in the metro area, living in a city predicted odds of same-sex cohabitation, OR 2.52, p<0.001, in 2000 data (p. 201)<br>• **In unadjusted models predicting odds of urban residence, young white same-sex couples had 8.58 times the likelihood of living in an urban area than white heterosexual couples, p<0.001, in 1990 data (p. 198)**
4	(Smart & Klein, 2013)	Cross-sectional, 2009 (and 2005-2009 ACS data), 11,450 individuals in opposite-sex couples and 802 individuals same-sex couples	USA, probability-based (National Household Transportation Survey), same-sex couples	USA, Census tracts	t test or pr test	• Comparing male and female same-sex households at the census tract level, the average number of people per square mile in the tract was not different for male same-sex couples than female same-sex couples, p<0.01<br>• Comparing same-sex to opposite-sex households at the census tract level, the average % housing built before 1950 in the tract was higher for male same-sex couples than opposite-sex couples, p<0.05<br>• **Comparing same-sex to opposite-sex households at the census tract level, the average number of jobs per square mile in the tract was marginally higher for male same-sex couples than opposite-sex couples, p<0.10**<br>• **Comparing same-sex to opposite-sex households at the census tract level, the average number of jobs per square mile in the tract was not different for female same-sex couples than opposite-sex couples, p>0.10**<br>• **Comparing same-sex to opposite-sex households at the census tract level, the average number of people per square mile in the tract was higher for male same-sex couples than opposite-sex couples, p<0.05**<br>• **Comparing same-sex to opposite-sex households at the census tract level, the average number of people per square mile in the tract was lower for female same-sex couples than opposite-sex couples, p>0.10**<br>• Comparing same-sex to opposite-sex households at the census tract level, the housing built before 1950s in the tract was higher for female same-sex couples than opposite-sex couples, p>0.10<br>• **Comparing male same-sex couples at the census tract level to female same-sex couples, there was no difference in the average number of jobs per square mile in the tract, p>0.10**<br>• Comparing male same-sex couples at the census tract level to

RoB*	Author 1 Year	Study Design, Year, N of individuals/couples	Sampling Frame: Sampling Strategy; definition of sexual orientation	Study area [Country: Location, Area Unit(s) of Analysis, N of area units]	Type of Quantitative Analysis Utilized	**Outcome(s)** (Bold indicates inclusion in Harvest Plot)
						female same-sex couples, there was there was no difference in the % of same-sex couples, p>0.10<br>• **Comparing same-sex to opposite sex households at the census tract level, the % of same-sex couples was higher for male same-sex couples than for opposite-sex couples, p<0.01**<br>• **Comparing same-sex to opposite sex households at the census tract level, the % of same-sex couples was not different for female same-sex couples than for opposite-sex couples, p>0.10**<br>• Comparing same-sex to opposite-sex households at the census tract level, the % housing built in 1950s in the tract was not different for male same-sex couples than female same-sex couples, p>0.10<br>• In the NHTS data, there is a general pattern of partnered gay and lesbians being in more urban areas.
4	(Smith, Rissel, Richters, Grulich, & Visser, 2003)	Cross-sectional, 2001-2002, 19307	Australian Adults aged 16-59: Probability-based; sexual identity, attraction, behavior	Australia, postcodes, NR	Descriptive	• **Men living in major cities were significantly more likely to report homosexual identity, p<0.001**<br>• **Women living in major cities were significantly more likely to report lesbian or bisexual identity, p=0.032**
4	(Spring, 2013)	Repeated Cross Sectional: 2000 & 2010 Census data, NR	U.S. Population: Census; derived same-sex household (unmarried same-sex cohabitation)	U.S. (100 most populous places), census places, 100	OLS regressions à characteristic predicting segregation of same-sex households compared to different-sex households	• In an adjusted model predicting 2010 female same-sex couple segregation in census places, the percent of the count of hate crimes based on sexual orientation was not associated with segregation, b=-0.02, p>0.05<br>• In an adjusted model predicting 2010 female same-sex couple segregation in census places, the percent of the population with a graduate degree was not associated with segregation, b=-0.16, p>0.05<br>• In an adjusted model predicting 2010 male same-sec couple segregation in census places, the percent new housing units was not associated with segregation, b=-0.19, p>0.05<br>• In an adjusted model predicting 2010 male same-sex couple segregation in census places, the percent of the count of hate crimes based on sexual orientation was not associated with segregation, b=-0.04, p>0.05<br>• **The % of census place residents with a graduate degree was positively associated with the proportion of households who were male same-sex couples, r=0.65, p<0.05**<br>• **The median home value in a census place was positively associated with the proportion of households who were male same-sex couples, r=0.62, p<0.05**<br>• In an adjusted model predicting 2010 male same-sec couple segregation in census places, the percent of households who were female same-sex couples was not associated with segregation, b=-

RoB*	Author 1 Year	Study Design, Year, N of individuals/couples	Sampling Frame: Sampling Strategy; definition of sexual orientation	Study area [Country: Location, Area Unit(s) of Analysis, N of area units]	Type of Quantitative Analysis Utilized	**Outcome(s)** (Bold indicates inclusion in Harvest Plot)
						2.67, p>0.05<br>• In an adjusted model predicting 2010 male same-sex couple segregation in census places, the percent of the population with a graduate degree was negatively associated with segregation, b=-0.48, p<0.05<br>• In an adjusted model predicting 2010 male same-sec couple segregation in census places, the median home value was not associated with segregation, b=0.11, p>0.05<br>• In an adjusted model, place % new housing units negatively associated with male same-sex partner segregation (b=-0.47).<br>• The change in census place segregation for female same-sex couples from 2000 to 2010 was not associated with the proportion of households who were female same-sex households, r=-0.04, p>0.05<br>• In an adjusted model predicting 2010 female same-sec couple segregation in census places, the median home value was positively associated with segregation, b=0.15, p<0.05<br>• **The # of hate crime reports submitted 2005-2009 in a census place was positively associated with the proportion of households who were female same-sex couples, r=0.20, p<0.05**<br>• **The % new housing units in a census place was not associated with the proportion of households who were female same-sex couples, r=-0.07, p>0.05**<br>• **The % of census place residents with a graduate degree was positively associated with the proportion of households who were female same-sex couples, r=0.44, p<0.05**<br>• **The # of hate crime reports submitted 2005-2009 in a census place was positively associated with the proportion of households who were male same-sex couples, r=0.24, p<0.05**<br>• **The % new housing units in a census place was not associated with the proportion of households who were male same-sex couples, r=-0.14, p>0.05**<br>• The change in census place segregation male same-sex couples from 2000 to 2010 was not associated with the proportion of households who were male same-sex households, r=-0.07, p>0.05<br>• In an adjusted model predicting 2010 female same-sec couple segregation in census places, the count of hate crime reports submitted was positively associated with segregation, b=0.23, p<0.01<br>• In an adjusted model predicting 2010 male same-sec couple segregation in census places, the count of hate crime reports submitted was not associated with segregation, b=0.07, p>0.05

RoB *	Author 1 Year	Study Design, Year, N of individuals/couples	Sampling Frame: Sampling Strategy; definition of sexual orientation	Study area [Country: Location, Area Unit(s) of Analysis, N of area units]	Type of Quantitative Analysis Utilized	**Outcome(s)** (Bold indicates inclusion in Harvest Plot)
						• In an adjusted model predicting 2010 male same-sec couple segregation in census places, the percent new housing units was not associated with segregation, b=-0.09, p>0.05<br>• In an adjusted model predicting 2010 male same-sec couple segregation in census places, the percent of households who were male same-sex couples was not associated with segregation, b=2.81, p>0.05<br>• **The # of hate crimes (2005-2009) in a census place was not associated with the proportion of households who were male same-sex couples, r=0.20, p>0.05**<br>• **The # of hate crimes reported in 2005-2009 in a census place was not associated with the proportion of households who were female same-sex couples, r=-0.10, p>0.05**<br>• **The median home value in a census place was positively associated with the proportion of households who were female same-sex couples, r=0.23, p<0.05**
4	(Wadsworth, Hickman, Johnson, Wellings, & Field, 1996)	Cross-sectional, 1990-1991, 18876	Age 16-59: Probability-based, sexual behavior	Great Britain, NR but likely approximate cities, NR	Descriptive	• **For women, a general pattern showed more women in urban areas had same-sex partners OR for inner London vs. growing/prosperous areas, 4.10 (1.64-10.2), p<0.05**<br>• For women, a general pattern showed more women in urban areas had same-sex partners OR for university/resort vs. growing/prosperous areas, 5.23 (2.19-12.50), p<0.05<br>• For men, a general pattern showed more men in urban areas reported ≥1 same-sex partner(s), OR for mining/industrial areas vs. growing/prosperous areas, 1.01 (0.52-2.00), p>0.05<br>• For men, a general pattern showed more men in urban areas reported ≥1 same-sex partner(s), OR for rural vs. growing/prosperous areas, 0.59 (0.25-1.40), p>0.05<br>• For men, a general pattern showed more men in urban areas reported ≥1 same-sex partner(s), OR for university/resort vs. growing/prosperous areas, 1.35 (0.59-3.07), p>0.05<br>• For women, a general pattern showed more women in urban areas had same-sex partners OR for outer London vs. growing/prosperous areas, 0.90 (0.23-3.58), p>0.05<br>• **For men, a general pattern showed more men in urban areas reported ≥1 same-sex partner(s), OR for inner London vs. growing/prosperous areas, 5.58 (3.10-10.0), p<0.05**<br>• For men, a general pattern showed more men in urban areas reported ≥1 same-sex partner(s), OR for Outer London vs. growing/prosperous areas, 1.62 (0.78-3.37), p>0.05<br>• For men, a general pattern showed more men in urban areas reported ≥1 same-sex partner(s), OR for urban manufacturing vs.

RoB*	Author 1 Year	Study Design, Year, N of individuals/couples	Sampling Frame: Sampling Strategy; definition of sexual orientation	Study area [Country: Location, Area Unit(s) of Analysis, N of area units]	Type of Quantitative Analysis Utilized	**Outcome(s)** (Bold indicates inclusion in Harvest Plot)
						growing/prosperous areas, 1.13 (0.57-2.21), p>0.05<br>• For women, a general pattern showed more women in urban areas had same-sex partners OR for mining/industrial areas vs. growing/prosperous areas, 1.45 (0.58-3.61), p>0.05<br>• For women, a general pattern showed more women in urban areas had same-sex partners OR for rural areas vs. growing/prosperous areas, 1.46 (0.54-3.96), p>0.05<br>• For women, a general pattern showed more women in urban areas had same-sex partners OR for urban/manufacturing vs. growing/prosperous areas, 0.69 (0.22-2.19), p>0.05
4	(Walther & Poston Jr, 2004)	Cross-sectional, 1990, NR	US households: Census (PUMS 5%); same-sex coupled household	USA: MSAs with >500,000 population, MSAs, 83	Correlation, linear regression	• **At the MSA level, index of partnered gay men was not associated the presence of a sodomy law covering both homosexual and heterosexual sex, r=-0.20, p=0.07**<br>• At the MSA level, index of lesbian women was negatively associated with a measure of July temperature, r=-0.48, p<0.001<br>• **At the MSA level, index of partnered gay men was negatively associated with % Republication, r=-0.35, p=0.001**<br>• **At the MSA level, index of partnered lesbian women was negatively associated with % Southern Baptist, r=-0.27, p=0.01**<br>• In an adjusted model at the MSA level, a measure of July temperatures was negatively associated with an index of coupled gay males, b=-0.58, p=0.03<br>• In an adjusted model at the MSA level, the % Southern Baptist was not associated with an index of coupled lesbian women, b=-0.00, p=0.82<br>• At the MSA level, index of gay men was negatively associated with a measure of July temperature, r=-0.35, p=0.001<br>• In an adjusted model at the MSA level, a measure of July temperatures was negatively associated with an index of coupled lesbian women, b=-0.44, p=0.001<br>• In an adjusted model at the MSA level, the % Republican was negatively associated with an index of coupled gay males, b=-0.34, p=0.02<br>• In an adjusted model at the MSA level, the % Republican was not associated with an index of coupled lesbian women, b=-0.18, p=0.12<br>• In an adjusted model at the MSA level, the % Southern Baptist was not associated with an index of coupled gay males, b=-0.01, p=0.81<br>• In an adjusted model at the MSA level, the presence of a sodomy law covering homosexual sex was not associated with an index of coupled gay males, b=1.10, p=0.79

RoB*	Author 1 Year	Study Design, Year, N of individuals/couples	Sampling Frame: Sampling Strategy; definition of sexual orientation	Study area [Country: Location, Area Unit(s) of Analysis, N of area units]	Type of Quantitative Analysis Utilized	**Outcome(s)** (Bold indicates inclusion in Harvest Plot)
						• In an adjusted model at the MSA level, the unemployment rate was negatively associated with an index of coupled gay males, b=-1.52, p=0.01<br>• In an adjusted model at the MSA level, the unemployment rate was negatively associated with an index of coupled lesbian women, b=-0.66, p=0.02<br>• **At the MSA level, index of partnered gay men was not associated the presence of a sodomy law covering only homosexual sex, r=-0.07, p=0.54**<br>• **At the MSA level, index of partnered gay men was not associated with the unemployment rate, r=-0.15, p=0.16**<br>• **At the MSA level, index of partnered lesbian women was negatively associated with % Republication, r=-0.41, p<0.001**<br>• **At the MSA level, index of partnered lesbian women was not associated the presence of a sodomy law covering only homosexual sex, r=-0.12, p=0.28**<br>• **At the MSA level, index of partnered lesbian women was negatively associated the presence of a sodomy law covering both homosexual and heterosexual sex, r=-0.29, p=0.01**<br>• **At the MSA level, index of partnered lesbian women was not associated with the unemployment rate, r=-0.48, p<0.001**<br>• **At the MSA level, index of partnered gay men was not associated with % Southern Baptist, r=-0.21, p=0.05**<br>• In an adjusted model at the MSA level, the presence of a sodomy law covering homosexual sex was not associated with an index of coupled lesbian women, b=0.75, p=0.71
4	(Walther, Poston Jr, & Gu, 2011)	Cross-sectional, 2000, NR	USA: Census; same-sex couple households	USA, MSA, 331	Correlation, regression	• In an adjusted model at the MSA level, better climate was positively associated with the gay male household rate, b=6.39, p<0.10<br>• In an adjusted model at the MSA level, hetero/homo-sexual rate was positively associated with the gay male household rate, b=0.02, p<0.10<br>• In an adjusted model at the MSA level, infant mortality was not associated with the lesbian household rate, b=-0.03, p>0.10<br>• In an adjusted model at the MSA level, unemployment was negatively associated with the gay male household rate, b=-0.13, p<0.10<br>• In an adjusted model at the MSA level, unemployment was negatively associated with the lesbian household rate, b=-0.15, p<0.10<br>• In an adjusted model at the MSA level, a measure of discrimination was negatively associated with the gay male household rate, b=-0.17, p<0.10

RoB*	Author 1 Year	Study Design, Year, N of individuals/couples	Sampling Frame: Sampling Strategy; definition of sexual orientation	Study area [Country: Location, Area Unit(s) of Analysis, N of area units]	Type of Quantitative Analysis Utilized	**Outcome(s)** (Bold indicates inclusion in Harvest Plot)
						• In an adjusted model at the MSA level, a measure of discrimination was negatively associated with the lesbian household rate, b=-0.18, p<0.10<br>• In an adjusted model at the MSA level, conservatism was negatively associated with the gay male household rate, b=-0.22, p<0.10<br>• In an adjusted model at the MSA level, infant mortality was not associated with the gay male household rate, b=0.01, p>0.10<br>• In an adjusted model at the MSA level, log of population size was positively associated with the gay male household rate, b=0.57, p<0.10<br>• At the MSA level, the partnered gay and partnered lesbian prevalence rates were positively correlated, r=0.69, p=NR<br>• In an adjusted model at the MSA level, better climate was positively associated with the lesbian household rate, b=5.09, p<0.10<br>• In an adjusted model at the MSA level, log of population size was positively associated with the lesbian household rate, b=0.18, p<0.10<br>• In an adjusted model at the MSA level, poverty was not associated with the gay male household rate, b=0.03, p>0.10<br>• In an adjusted model at the MSA level, poverty was not associated with the lesbian household rate, b=0.05, p>0.10<br>• In an adjusted model at the MSA level, conservatism was negatively associated with the lesbian household rate, b=-0.22, p<0.10<br>• In an adjusted model at the MSA level, hetero/homo-sexual rate was positively associated with the lesbian household rate, b=0.05, p<0.10
3	(Wimark & Östh, 2014)	Cross-sectional, 2011, 66,393 LGB individuals in Qruiser web site and from same-sex marriage registry 6,549	Qruiser web site members; registered partnership/marriage registry: Census; self-identified (Qruiser) and same-sex couple (registry)	Sweden: municipalities, 290	Correlation, Regression	• The unadjusted correlation at the municipality level between the website-derived single and coupled male GB index and municipality population is positive, r=0.92<br>• The unadjusted correlation at the municipality level between the website-derived LGB population and municipality population is positive, r=0.92<br>• The unadjusted correlation at the municipality level between the website-derived single and coupled female LGB index and municipality population is positive, r=0.88<br>• The unadjusted correlation at the municipality level between the same-sex couple registry data and municipality population is positive, r=0.75<br>• The unadjusted correlation at the municipality level between the website-derived female couple index and municipality population is positive, r=0.84<br>• The unadjusted correlation at the municipality level between the

RoB*	Author 1 Year	Study Design, Year, N of individuals/couples	Sampling Frame: Sampling Strategy; definition of sexual orientation	Study area [Country: Location, Area Unit(s) of Analysis, N of area units]	Type of Quantitative Analysis Utilized	**Outcome(s)** (Bold indicates inclusion in Harvest Plot)
						website-derived index of LGB couples and municipality population size is positive, r=0.90<br>• Where urbanicity is concerned, data using same-sex couples may substantially underestimate the draw of more urban areas among single LGB people. The unadjusted correlation at the municipality level between the website-derived male-couple index and municipality population is positive, r=0.89
3	(Wimark, 2014)	Cross-sectional, 2011, N.R.	Sweden: Census, self-identification in Qruiser web site	Sweden: Labor Market Region, 75	Correlation	• **At the labor market region area unit, the gay index is not associated with a right-wing party voting, r=-0.038, p>0.10**<br>• **At the labor market region area unit, the gay index is not associated with integration, r=0.074, p>0.10**<br>• **At the labor market region area unit, the gay index is not associated with hate crimes, r=0.129, p>0.10**<br>• **At the labor market region area unit, the lesbian index is not associated with a right-wing party voting, r=-0.007, p>0.10**<br>• In an adjusted regression model at the labor market region area unit, the gay index is not associated with income after control for population size and educational attainment, b=-0.001, p>0.10<br>• **At the labor market region area unit, the gay index is positively associated with a measure of technology, r=0.831, p<0.001**<br>• **At the labor market region area unit, the gay index is positively associated with the bohemian index, r=0.703, p<0.001**<br>• **At the labor market region area unit, the lesbian index is positively associated with a measure of technology, r=0.743, p<0.001**<br>• In an adjusted regression model at the labor market region area unit, the gay index is associated with creative class, b=0.016, p<0.001<br>• In an adjusted regression model at the labor market region area unit, the gay index is not associated with income after control for population size, b=0.006, p>0.10<br>• **In an unadjusted regression model at the labor market region area unit, the gay index is significantly associated with income, b=0.031, p<0.001**<br>• **At the labor market region area unit, the gay index is positively associated with creative class, r=0.773, p<0.001**<br>• **At the labor market region area unit, the lesbian index is positively associated with creative class, r=0.712, p<0.001**<br>• **At the labor market region area unit the gay index is associated with the lesbian index, r=0.786, p<0.001**<br>• **At the labor market region area unit, the gay index is positively associated with income, r=0.638, p<0.001**

RoB	Author 1 Year	Study Design, Year, N of individuals/couples	Sampling Frame: Sampling Strategy; definition of sexual orientation	Study area [Country: Location, Area Unit(s) of Analysis, N of area units]	Type of Quantitative Analysis Utilized	**Outcome(s)** (Bold indicates inclusion in Harvest Plot)
						• **At the labor market region area unit, the lesbian index is not associated with integration, r=-0.049, p>0.10**<br>• **At the labor market region area unit, the lesbian index is positively associated with the melting pot index, r=0.484, p<0.001**<br>• **At the labor market region area unit, the gay index is associated with the melting pot index, r=0.527, p<0.001**<br>• **At the labor market region area unit, the lesbian index is positively associated with income, r=0.511, p<0.001**<br>• **At the labor market region area unit, the lesbian index is positively associated with the bohemian index, r=0.590, p<0.001**<br>• In an adjusted regression model at the labor market region area unit, the gay index is positively associated with creative class after control for population size, b=0.007, p<0.001<br>• **At the labor market region area unit, the lesbian index is not associated with hate crimes, r=0.104, p>0.10**<br>• In an adjusted regression model at the labor market region area unit, the gay index is not associated with creative class after control for population size and education, b=0.00, p>0.10

Note: RoB = Risk of Bias Index (0 = High Risk of Bias, 4 = Low Risk of Bias)

References

Adler, S. Y., & Brenner, J. (1992). Gender and Space: Lesbian and Gay Men in the City. *International Journal of Urban & Regional Research, 16*(1), 24. doi:10.1111/j.1468-2427.1992.tb00463.x

Anacker, K. B., & Morrow-Jones, H. A. (2005). Neighborhood factors associated with same-sex households in US cities. *Urban Geography, 26*(5), 385-409. doi:10.2747/0272-3638.26.5.385

Andersson, G., Noack, T., Seierstad, A., & Weedon-Fekjær, H. (2006). The Demographics of Same-Sex Marriages in Norway and Sweden. *Demography, 43*(1), 79-98. doi:10.1353/dem.2006.0001

Bailey, R. W. (1999). Identity, urban space, and political action. In (pp. 49-95). New York: Columbia University Press.

Bauermeister, J. A., Eaton, L., Andrzejewski, J., Loveluck, J., VanHemert, W., & Pingel, E. S. (2015). Where You Live Matters: Structural Correlates of HIV Risk Behavior Among Young Men Who Have Sex with Men in Metro Detroit. *AIDS and behavior, 19*(12), 2358-2369. doi:10.1007/s10461-015-1180-1

Baumle, A. K. (2010). Border identities: Intersections of ethnicity and sexual orientation in the U.S.–Mexico borderland. *Social science research, 39*(2), 231-245. doi:10.1016/j.ssresearch.2009.08.005

Baumle, A. K., Compton, D. R., & Poston, D. L. (2009a). Patterns of same-sex partnering in metropolitan and nonmetropolitan America. In (pp. 41-58). Albany: SUNY Press.

Baumle, A. K., Compton, D. R., & Poston, D. L. (2009b). The residential segregation of gay males and lesbians from heterosexuals. In (pp. 59-71). Albany: SUNY Press.

Bennett, K., McElroy, J. A., Johnson, A. O., Munk, N., & Everett, K. D. (2015). A Persistent Disparity: Smoking in Rural Sexual and Gender Minorities. *LGBT Health, 2*(1), 62-70. doi:10.1089/lgbt.2014.0032

Bereitschaft, B., & Cammack, R. (2015). Neighborhood diversity and the creative class in Chicago. *Applied Geography, 63*, 166-183. doi:10.1016/j.apgeog.2015.06.020

Binson, D., Michaels, S., Stall, R., Coates, T. J., Gagnon, J. H., & Catania, J. A. (1995). Prevalence and social distribution of men who have sex with men: United States and its urban centers. *Journal of sex research, 32*(3), 245-254. doi:10.1080/00224499509551795

Black, D., Gates, G., Sanders, S., & Taylor, L. (2002). Why do gay men live in San Francisco? *Journal of Urban Economics, 51*(1), 54-76. doi:10.1006/juec.2001.2237
Chen, X. (2011). Tolerance and Economic Performance in American Metropolitan Areas: An Empirical Investigation. *Sociological Forum, 26*(1), 71-97. doi:10.1111/j.1573-7861.2010.01225.x
Chen, X., Yu, B., Zhou, D., Zhou, W., Gong, J., Li, S., & Stanton, B. (2015). A Comparison of the Number of Men Who Have Sex with Men among Rural-To-Urban Migrants with Non-Migrant Rural and Urban Residents in Wuhan, China: A GIS/GPS-Assisted Random Sample Survey Study. *PloS one, 10*(8), e0134712. doi:10.1371/journal.pone.0134712
Christafore, D., Leguizamon, J. S., & Leguizamon, S. (2013). Are black neighborhoods less welcoming to homosexuals than white neighborhoods? *Regional Science and Urban Economics, 43*(4), 579-589. doi:10.1016/j.regsciurbeco.2013.02.006
Christafore, D., & Leguizamon, S. (2012). The influence of gay and lesbian coupled households on house prices in conservative and liberal neighborhoods. *Journal of Urban Economics, 71*(2), 258-267. doi:10.1016/j.jue.2011.09.004
Collins, T. W., Grineski, S. E., & Morales, D. X. (2016). Sexual Orientation, Gender, and Environmental Injustice: Unequal Carcinogenic Air Pollution Risks in Greater Houston. *Annals of the American Association of Geographers*(Journal Article), 1-21. doi:10.1080/24694452.2016.1218270
Compton, D. R., & Baumle, A. K. (2012). Beyond the Castro: the role of demographics in the selection of gay and lesbian enclaves. *J Homosex, 59*(10), 1327-1355. doi:10.1080/00918369.2012.724633
Cooke, T. J. (2005). Migration of same-sex couples. *Population, Space and Place, 11*(5), 401-409. doi:10.1002/psp.383
Cooke, T. J., & Rapino, M. (2007). The migration of partnered gays and lesbians between 1995 and 2000. *Professional Geographer, 59*(3), 285-297. doi:10.1111/j.1467-9272.2007.00613.x
Croes, M. M. (1996). [Same-sex cohabitation]. *Maandstat Bevolking, 44*(10), 24-26.
Doan, P., & Higgins, H. (2009). Cognitive dimensions of way-finding: the implications of habitus, safety, and gender dissonance among gay and lesbian populations. *Environment and Planning A, 41*(7), 1745-1762. doi:10.1068/a4159
Elder, G., Rothblum, E. D., & Solomon, S. E. (2010). The geography of civil union households. *Journal of GLBT Family Studies, 6*(1), 58-67. doi:10.1080/15504280903472519
Ernst, R. S., & Houts, P. S. (1985). Characteristics of gay persons with sexually transmitted disease. *Sexually transmitted diseases, 12*(2), 59-63.
Everett, B. G. (2014). Changes in Neighborhood Characteristics and Depression Among Sexual Minority Young Adults. *Journal of the American Psychiatric Nurses Association, 20*(1), 42-52. doi:10.1177/1078390313510319
Fanning, J. M., & Ruther, M. (2015). Gayborhoods: Economic development and the concentration of same-sex couples in neighborhoods within large American cities. In P. Nijkamp, A. Rose, & K. Kourtit (Eds.), *Regional Science Matters* (pp. 399-420). Switzerland: Springer.
Farmer, G. W., Blosnich, J. R., Jabson, J. M., & Matthews, D. D. (2016). Gay Acres: Sexual Orientation Differences in Health Indicators Among Rural and Nonrural Individuals. *Journal of Rural Health, 32*(3), 321-331. doi:10.1111/jrh.12161
Fasula, A. M., Oraka, E., Jeffries, W. L. t., Carry, M., Banez Ocfemia, M. C., Balaji, A. B., . . . Reproductive, H. (2016). Young Sexual Minority Males in the United States: Sociodemographic Characteristics And Sexual Attraction, Identity and Behavior. *Perspectives on sexual and reproductive health, 48*(1), 3-8. doi:10.1363/48e7016 [doi]
Florida, R. (2002). The economic geography of talent. *Annals of the American Association of Geographers, 92*(4), 743-755. doi:10.1111/1467-8306.00314
Gates, G. J. (2013). Demographic perspectives on sexual orientation. In C. J. Patterson & A. R. D'Augelli (Eds.), *Handbook of psychology and sexual orientation* (pp. 69-84). New York, NY, US: Oxford University Press.
Gates, G. J., & Ost, J. (2004). *The gay & lesbian atlas*. Washington, D.C: Urban Institute Press.
Giraud, C. (2011). Residential Spaces: Looking at Where Homosexual Men in Paris Choose to Live. *Societes Contemporaines, 81*(1), 151-176. doi:http://dx.doi.org/10.3917/soco.081.0151
Guo, C., Pang, L., Zhang, L., Chen, G., Wang, Z., & Zheng, X. (2016). Disparities of sexual orientations by sex and urban or rural residence among youth in China. *Sexual health*(Journal Article). doi:10.1071/SH16041 [doi]
Hayslett, K. L., & Kane, M. D. (2011). "Out" in Columbus: A Geospatial Analysis of the Neighborhood-Level Distribution of Gay and Lesbian Households. *City and Community, 10*(2), 131-156. doi:10.1111/j.1540-6040.2010.01353.x
Hughes, A., & Saxton, P. (2006). Geographic Micro-Clustering of Homosexual Men: Implications for Research and Social Policy. *Social Policy Journal of New Zealand/Te Puna Whakaaro*(28), 158-178.
Klein, N. J., & Smart, M. J. (2016). Travel mode choice among same-sex couples. *Transportation Research Part A: Policy & Practice, 90*(Journal Article), 1-13.
Laumann, E. O., Gagnon, J. H., Michael, R. T., & Michaels, S. (1994). Homosexuality. In *The Social Organization of Sexuality* (pp. 283-320). Chicago: University of Chicago Press.

Lee, J. G., Goldstein, A. O., Pan, W. K., & Ribisl, K. M. (2015). Relationship Between Tobacco Retailers' Point-of-Sale Marketing and the Density of Same-Sex Couples, 97 US Counties, 2012. *International Journal of Environmental Research and Public Health, 12*(8), 8790-8810. doi:10.3390/ijerph120808790

Lee, J. G., Pan, W. K., Henriksen, L., Goldstein, A. O., & Ribisl, K. M. (2016). Is There a Relationship Between the Concentration of Same-Sex Couples and Tobacco Retailer Density? *Nicotine & tobacco research, 18*(2), 147-155. doi:10.1093/ntr/ntv046

Marcus, U., Schmidt, A. J., Hamouda, O., & Bochow, M. (2009). Estimating the regional distribution of men who have sex with men (MSM) based on Internet surveys. *BMC Public Health, 9*, 180. doi:10.1186/1471-2458-9-180

Matthews, P., & Besemer, K. (2015). The “Pink Pound” in the “Gaybourhood”? Neighbourhood Deprivation and Sexual Orientation in Scotland. *Housing, Theory & Society, 32*(1), 94-111. doi:10.1080/14036096.2014.991809

Minnis, A. M., Catellier, D., Kent, C., Ethier, K. A., Soler, R. E., Heirendt, W., . . . Rogers, T. (2016). Differences in chronic disease behavioral indicators by sexual orientation and sex. *Journal of Public Health Management and Practice, 22*, S25-S32. doi:10.1097/PHH.0000000000000350

Rosenfeld, M. J. (2007). *The age of independence: interracial unions, same-sex unions, and the changing American family*. Cambridge, Mass: Harvard University Press.

Smart, M. J., & Klein, N. J. (2013). Neighborhoods of Affinity Social Forces and Travel in Gay and Lesbian Neighborhoods. *Journal of the American Planning Association, 79*(2), 110-124. doi:10.1080/01944363.2013.883227

Smith, A. M. A., Rissel, C. E., Richters, J., Grulich, A. E., & Visser, R. O. (2003). Sex in Australia: Sexual identity, sexual attraction and sexual experience among a representative sample of adults. *Australian and New Zealand Journal of Public Health, 27*(2), 138-145. doi:10.1111/j.1467-842X.2003.tb00801.x

Spring, A. L. (2013). Declining Segregation of Same-Sex Partners: Evidence from Census 2000 and 2010. *Population Research and Policy Review, 32*(5), 687-716. doi:10.1007/s11113-013-9280-y

Wadsworth, J., Hickman, M., Johnson, A. M., Wellings, K., & Field, J. (1996). Geographic variation in sexual behaviour in Britain: Implications for sexually transmitted disease epidemiology and sexual health promotion. *AIDS, 10*(2), 193-199. doi:10.1097/00002030-199602000-00010

Walther, C. S., & Poston Jr, D. L. (2004). Patterns of gay and lesbian partnering in the larger metropolitan areas of the United States. *Journal of sex research, 41*(2), 201-214.

Walther, C. S., Poston Jr, D. L., & Gu, Y. (2011). Ecological Analyses of Gay Male and Lesbian Partnering in the Metropolitan United States in 2000. *Population Research and Policy Review, 30*(3), 419-448. doi:10.1007/s11113-010-9195-9

Wimark, T. (2014). Is it really tolerance? Expanding the knowledge about diversity for the creative class. *Tijdschrift voor economische en sociale geografie, 105*(1), 46-63. doi:10.1111/tesg.12044

Wimark, T., & Östh, J. (2014). The City as a Single Gay Male Magnet? Gay and Lesbian Geographical Concentration in Sweden. *Population, Space and Place, 20*(8), 739-752. doi:10.1002/psp.1825
